# Genome-wide identification and expression profiling of serine proteases and homologs in the diamondback moth, *Plutella xylostella* (L.)

**DOI:** 10.1186/s12864-015-2243-4

**Published:** 2015-12-10

**Authors:** Hailan Lin, Xiaofeng Xia, Liying Yu, Liette Vasseur, Geoff M. Gurr, Fengluan Yao, Guang Yang, Minsheng You

**Affiliations:** Institute of Applied Ecology and Research Centre for Biodiversity and Eco-Safety, Fujian Agriculture and Forestry University, Fuzhou, 350002 China; College of Life Science, Fujian Agriculture and Forestry University, Fuzhou, 350002 China; Fujian-Taiwan Joint Centre for Ecological Control of Crop Pests, Fujian Agriculture and Forestry University, Fuzhou, 350002 China; Key Laboratory of Integrated Pest Management of Fujian and Taiwan, China Ministry of Agriculture, Fuzhou, 350002 China; Department of Biological Sciences, Brock University, 1812 Sir Isaac Brock Way, St. Catharines, ON L2S 3A1 Canada; Graham Centre, Charles Sturt University, Orange, NSW 2800 Australia; Institute of Plant Protection, Fujian Academy of Agricultural Sciences, Fuzhou, 350013 China

**Keywords:** Digestive enzyme, Expression pattern, Trypsin, Chymotrypsin, Lepidoptera

## Abstract

**Background:**

Serine proteases (SPs) are crucial proteolytic enzymes responsible for digestion and other processes including signal transduction and immune responses in insects. Serine protease homologs (SPHs) lack catalytic activity but are involved in innate immunity. This study presents a genome-wide investigation of SPs and SPHs in the diamondback moth, *Plutella xylostella* (L.), a globally-distributed destructive pest of cruciferous crops.

**Results:**

A total of 120 putative SPs and 101 putative SPHs were identified in the *P. xylostella* genome by bioinformatics analysis. Based on the features of trypsin, 38 SPs were putatively designated as trypsin genes. The distribution, transcription orientation, exon-intron structure and sequence alignments suggested that the majority of trypsin genes evolved from tandem duplications. Among the 221 SP/SPH genes, ten SP and three SPH genes with one or more clip domains were predicted and designated as PxCLIPs. Phylogenetic analysis of CLIPs in *P. xylostella*, two other Lepidoptera species (*Bombyx mori* and *Manduca sexta*), and two more distantly related insects (*Drosophila melanogaster* and *Apis mellifera*) showed that seven of the 13 PxCLIPs were clustered with homologs of the Lepidoptera rather than other species. Expression profiling of the *P. xylostella* SP and SPH genes in different developmental stages and tissues showed diverse expression patterns, suggesting high functional diversity with roles in digestion and development.

**Conclusions:**

This is the first genome-wide investigation on the SP and SPH genes in *P. xylostella*. The characterized features and profiled expression patterns of the *P. xylostella* SPs and SPHs suggest their involvement in digestion, development and immunity of this species. Our findings provide a foundation for further research on the functions of this gene family in *P. xylostella*, and a better understanding of its capacity to rapidly adapt to a wide range of environmental variables including host plants and insecticides.

**Electronic supplementary material:**

The online version of this article (doi:10.1186/s12864-015-2243-4) contains supplementary material, which is available to authorized users.

## Background

Serine proteases (SPs) represent a very diverse group of proteolytic enzymes involved in digestion, development, and innate immunity [1–6]. X-ray crystal structural examination suggests that SPs possess a catalytic triad, consisting of His, Asp, and Ser amino acid residues [7], frequently embedded in the conserved sequences of TAAHC, DIAL, and GDSGGP, respectively [5, 8]. SPs commonly take the form of inactive pro-enzymes and require a specific and limited proteolytic cleavage for activation in a cascade pathway [8]. Extracellular serine proteinase cascades have evolved in invertebrates [9] to play critical roles in embryonic development and innate immunity [5, 6, 9] to mediate fast responses to infection and wounding [9]. A classical characteristic of these enzymes is that they have clip domain(s) at the amino terminus. Clip domains contain six conserved Cys residues with Cys-5 and Cys-6 at adjacent positions, forming three disulfide bonds [8, 9]. They may be involved in mediating protein-protein interactions or for regulating cascades of SP activities [6].

Serine protease homologs (SPHs) are also members of the SP family [5, 8, 10, 11] and have similar sequences to SPs, with the exception of mutations or absence of the catalytic residues, resulting in loss of catalytic function [8]. The roles of SPHs have been extensively studied in invertebrates. For example, SPHs are indispensable for activation of prophenoloxidase (proPO) in *Manduca sexta* (Lepidoptera: Sphingidae) [12–14]. SPHs are also involved in somatic muscle attachment in *Drosophila* (Diptera: Drosophilidae) embryos, regulation of complement recruitment to microbial surfaces in *Anopheles gambiae* (Diptera: Culicidae), cell adhesion in *Pacifastacus leniusculus* (Decapoda: Astacidae) and immune defense against bacterial infection in *Scylla paramamosain* (Decapoda: Portunidae) [15–18].

Development of DNA sequencing technologies has enabled whole-genome investigation of the SP and SPH genes in *Drosophila melanogaster*, *Apis mellifera* (Hymenoptera: Apidae), *Bombyx mori* (Lepidoptera: Bombycidae) and *Nilaparvata lugens* (Hemiptera: Delphacidae) [5, 8, 10, 11]. Further, immunity-related SPs and SPHs have been reported in *A. gambiae*, *A. mellifera*, *Tribolium castaneum* (Coleoptera: Tenebrionidae) and *B. mori* [5, 19–21]. Research of SPs and SPHs in these insect species has provided an overview of roles in triggering immunity responses.

The diamondback moth (DBM), *Plutella xylostella* (L.) (Lepidoptera: Plutellidae), is a devastating pest of cruciferous crops, costing an estimated $4–5 billion per annum around the world [22]. Populations of *P. xylostella* have been shown to commonly develop resistance to insecticides, including those based on the bacterium *Bacillus thuringiensis* (Bt), making it difficult to control [23]. Although the genome has been sequenced, and our recent work has identified 149 immune-related genes in *P. xylostella* immune system [24], the roles of SPs and SPHs in *P. xylostella* immunity and other physiological processes are not well understood. Only seven SPs have been reported with one chymotrypsin and three trypsins being cloned and downregulated in *P. xylostella* parasitized by *Cotesia vestalis* [25], and three clip serine proteases being identified and found to be associated with immunity [26].

In the present work, we identified and characterized the SP and SPH genes, and profiled their expression patterns in different life stages and tissues based on the *P. xylostella* genome (version 2, [27]), RNA-seq data and qPCR analysis. Our findings provide a foundation for further studies on biological functions of this gene family in *P. xylostella*, particularly associated with digestion, development and immunity.

## Results and discussion

### Identification and characterization of the *P. xylostella* SPs and SPHs

A total of 221 putative *P. xylostella* SPs and SPHs (PxSPs/PxSPHs) were identified in the *P. xylostella* genome (Additional file 1: Table S1). The protein sequences of 221 SP/SPH genes are provided in Additional file 2: Table S2. Based on the MEROPS process, the results showed that the majority of SPs/SPHs were significantly similar to the chymotrypsin (S1) family. Among the SP/SPH genes recognized, 82 were documented in 2013 when the *P. xylostella* genome was published [27]. The number of SP/SPH genes in *P. xylostella* is less than that in *A. gambiae* (306) [19], similar to that in *D. melanogaster* (204) [8], but greater than that in *B. mori* (143) [10], *N. lugens* (90) [11] and *A. mellifera* (57) [5].

According to the presence or absence of the catalytic triad, the 221 putative SP/SPH genes in *P. xylostella* were divided into 120 SP and 101 SPH genes (Additional file 1: Table S1). Of 120 PxSPs, 107 (89.2 %) contained an intact trypsin-like serine protease catalytic triad (Tryp_SPc) domain with the catalytic triad, while some had additional Tryp_SPc domains or other modules, including clip domain(s), low-density lipoprotein receptor class A (LDLA) domain, frizzled (FRI) domain and scavenger receptor Cys-rich (SR) domain (Additional file 1: Table S1). Aside from three SPHs (Px001667, Px011499 and Px013162) with an additional domain (clip domain) (Additional file 1: Table S1), the remaining SPHs had only the Tryp_SPc domain with one or more active sites replaced by other amino acid residues.

The 221 SP and SPH genes were spread across 119 different scaffolds (Additional file 1: Table S1), and 122 SP/SPH genes were predicted to be tandem duplications and located on 35 different scaffolds forming 36 clusters, each of which containing two or more 2 genes (Additional file 3: Figure S1). Eleven SP/SPH genes forming two clusters were located on scaffold 27, eight on scaffold 194, and seven on scaffolds 76 and 280 (Additional file 3: Figure S1). Similarly, large clusters of SP/SPH genes have been identified in the genomes of several species, such as *D. melanogaster*, *B. mori*, *N. lugens* and *A. gambiae*, representing different insect orders [8, 10, 11, 19]. Gene duplication and unequal crossing-over may be crucial mechanisms for production of large clusters [8]. It has also been suggested that large clusters of the SP and SPH genes from *B. mori* are tandem repeats [10]. Full chromosomal scaffolding information of *P. xylostella* will contribute to investigation of the PxSP and PxSPH duplication events, providing information on the evolution of this gene family. Based on the different functions of SPs and SPHs, SP/SPH genes were roughly classified into three major clades: 1) trypsin and chymotrypsin, 2) clip-domain SP/SPH and, 3) other SP/SPH genes.

### Trypsin and chymotrypsin genes

From the *P. xylostella* genome, we recognized 38 trypsin and 8 chymotrypsin genes (Additional file 1: Table S1). Both trypsin and chymotrypsin genes contain a relatively simple structure (Tryp_SPc) with the catalytic triad that characterizes all serine proteinases, and a typical substrate-binding pocket [5, 8]. Trypsin and chymotrypsin are well-studied serine proteases, playing vital roles in the digestion of proteins [28], as well as in the modulation of toxicity of Bt toxins [29, 30].

**Fig. 1:**
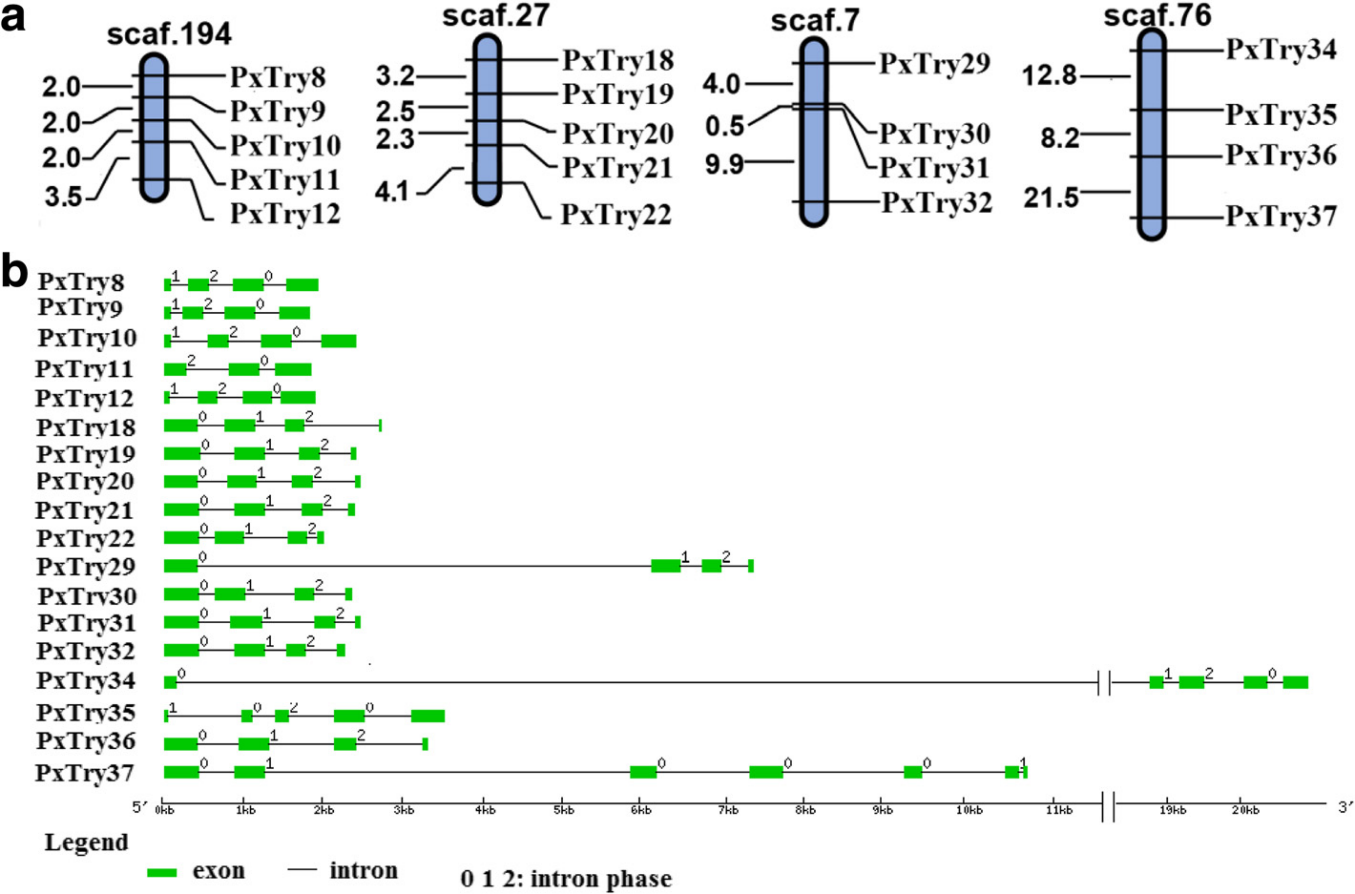
Scaffold localization and gene structure of *P. xylostella* trypsin genes. (**a**) The four scaffolds with ≥ four trypsin genes. Genes names and the distance of two adjacent genes (kilobases, kb) are showed on the right and left of the bar, respectively. Scaffold numbers are presented at the top of each bar. (**b**) Gene structure of the *P. xylostella* trypsins located on the four scaffolds was shown in Fig. 1a. Values (0, 1, and 2) above each of the black lines indicate the intron phase. The scale line at the bottom shows the length of each gene

The 38 trypsin genes were unevenly distributed among 23 different scaffolds (Additional file 1: Table S1). For example, scaffolds 194 and 27 had the largest trypsin clusters with five genes on each. Scaffolds 7 and 76 had four genes on each, while 18 scaffolds had only one gene each. Twenty trypsin genes formed 5 clusters on 5 scaffolds, which accounted for 52.6 % of all trypsin genes (Fig. 1a and Additional file 1: Table S1), and genes in each cluster were predicted to be tandem duplications. More specifically, PxTrys 8–10 had a high level of sequence similarity (85.1 %) and contained the same number of exons and intron phases (1-2-0) (Fig. 1b). The distances between PxTry8 and PxT ry9 as well as PxTry9 and PxTry10 are the same and only 2 kb (Fig. 1a). PxTrys 18–22 were on scaffold 27 with the same orientation and 80.1 % of the sequence similarity, and composed of the same number of exons and intron phases (0-1-2) (Fig. 1b). PxTrys 29–32 were on scaffold 7 with the same orientation, and shared 67.6 % of the sequence similarity and the same number of exons and intron phases (0-1-2) (Fig. 1b). Overall these results indicate that trypsin genes probably evolved from duplication events, like in *D. melanogaster* and *N. lugens* [8, 11]. The reasons for trypsins to duplicate in herbivorous insects remain unclear but two hypotheses have been proposed: a) increased expression of inhibitor-insensitive protease isoforms [31], and b) formation of a complex digestive system that provides an efficient mechanism for protein degradation [32, 33].

Multiple sequence alignment of *P. xylostella* trypsins along with well annotated trypsins, *Aedes aegypti* trypsin 3A1 (AaTry3A1), *A. gambie* trypsin-6 (AgTry6), and *Culex quinquefasciatus* trypsin1 (CqTry1) and trypsin5 (CqTry5), showed that *P. xylostella* trypsins shared features/domains with trypsins identified in other invertebrates (Additional file 4: Figure S2). As previously mentioned, they had the three motifs (THAAC, DIAL, and GDSGGP) as well as six cysteine (Cys) residues at conserved positions, and putative autocatalytic activation motifs. Thirty-eight putative *P. xylostella* trypsins had the characteristic Asp in the S1 pocket. However, some of the trypsins found in this study had other distinct characteristics. For instance, instead of Arg/Lys (R/K) residues, the autocatalytic motif of PxTry26, PxTry28, and PxTry34 had a Tyr (Y), Phe (F), and Y residues, respectively. This could indicate that they might be specific signals for activation. Moreover, the activation motif of PxTry14, PxTry24, PxTry28, and PxTry34 was IIGG, and IING for PxTry13 and PxTry26, different from the typical motif sequence (IVGG) [34, 35].

**Fig. 2:**
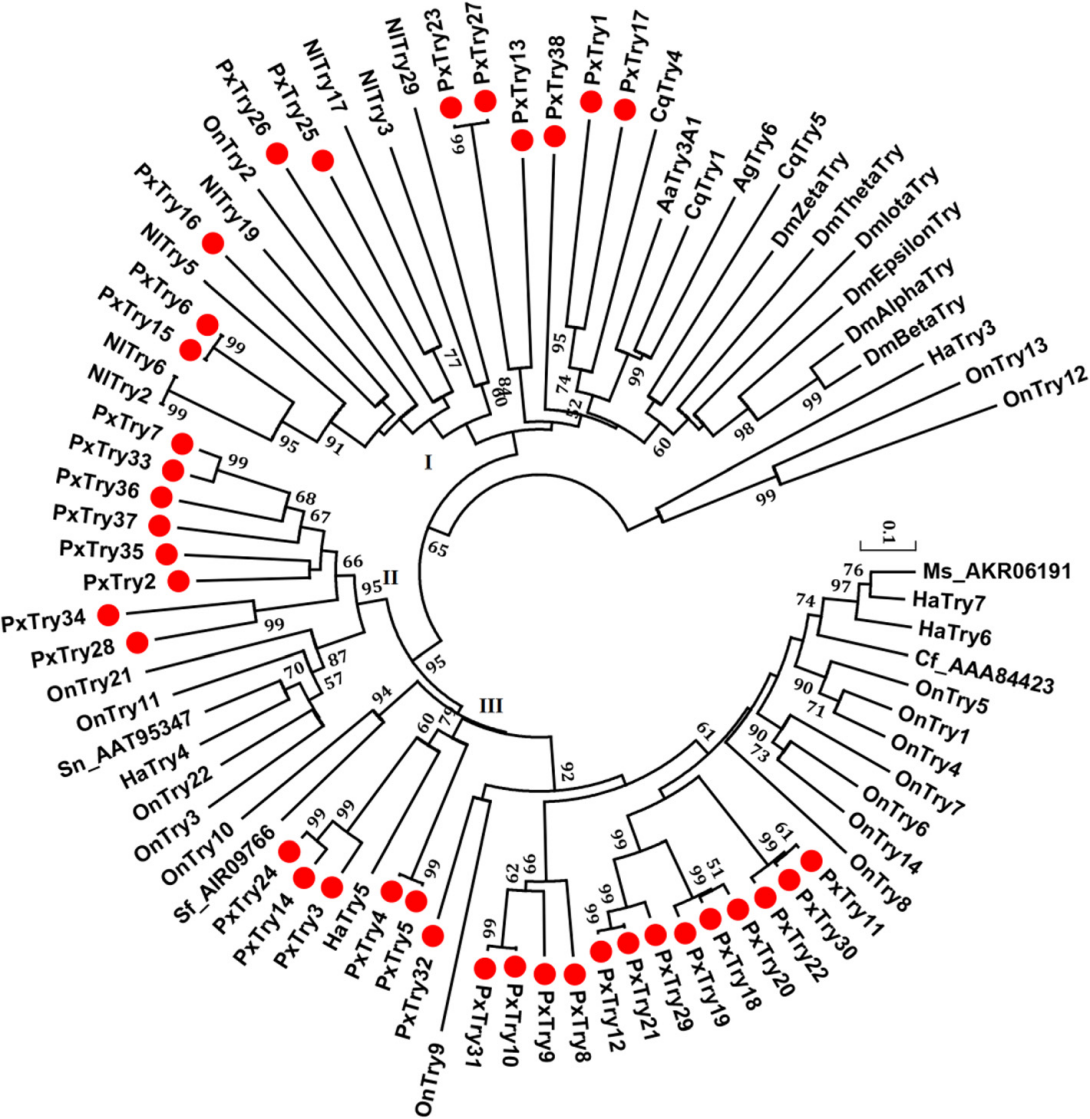
Phylogenetic relationship of trypsins in *P. xylostella* and other insect species. The phylogenetic tree was constructed using MEGA 6.06 with neighbor joining approach based on Poisson model and pairwise deletion of gaps. The percentage bootstrap scores higher than 50 % are indicated on the nodes. The first two letters in each of the gene names represent the acronym of scientific name for a given species (Cf: *Choristoneura fumiferana*; Sn: *Sesamia nonagrioides*; Sf*: Spodoptera frugiperda*; Ms: *Mythimna separate*; Aa: *Aedes aegypti*; Ag: *Anopheles gambiae*; Px: *Plutella xylostella*; Nl: *Nilaparvata lugens*; Cq: *Culex quinquefasciatus*; On*: Ostrinia nubilalis*; Dm: *Drosophila melanogaster*; Ha*: Helicoverpa armigera*). *P. xylostella* trypsins are marked with red dots

Phylogenetic analysis of trypsin genes showed that *P. xylostella* trypsins were clustered into three clades (I, II and III) (Fig. 2). Clade I contained 11 *P. xylostella* trypsin genes, which were clustered with those in *C. quinquefasciatus* and *N. lugens*. In this clade, CqTrys 1, 4, and 5 have been reported to be constitutively expressed in the midgut of females, indicating their potential roles in digestion [36]. NlTry2 and NlTry5 are highly expressed in the midgut [11]. Six *D. melanogaster* trypins, which were part of this Clade I, have been documented to play an important role in digestion [37].

Clade II contained eight *P. xylostella* trypsins, all of which were clustered together in a single branch. We predicted that PxTrys 34–37 were tandem duplications. In *Helicoverpa armigera*, HaTry4 is highly expressed in the larvae [38]. In *O. nubilalis*, OnTrys 3, 11, 22, and 21 are highly expressed in the midgut and hindgut [29]. Clade III contained 19 *P. xylostella* trypsins. As suggested by scaffold results, PxTrys 4–5, PxTrys 8–12, PxTrys 18–22 and PxTrys 29–32 were predicted to be tandem duplications. OnTrys 4–6 and 14 have been found to be up-regulated in *O. nubilalis* larvae after feeding on Cry1Ab protoxin [29]. Cry1Ab protoxin is one of the Cry toxins produced by the bacterium *B. thuringiensis*, which is used in biological insecticides applied to control pest insects in the fields [39].

The putative chymotrypsins have typical conserved sequence motifs, N-terminal putative activation residues (Arg or Lys) for cleavage and three catalytic triad residues (Additional file 5: Figure S3). However, PxChy8 had other distinct characteristic with Tyr (Y) instead of Arg/Lys (R/K) residues, suggesting a different specific signal for activation (Additional file 5: Figure S3 and Additional file 1: Table S1). The substrate binding pocket in chymotrypsins was relatively diverse, and PxChy2 and PxChy3 had the characteristic Ser in the S1 pocket, with the remaining being an Ala/Ser or Gly/Ser substituted in the S1 pocket. The Gly/Ser substitution in the S1 pocket has been found in *O. nubilalis* [29], and has presumably minor effects on substrate interactions.

**Fig. 3:**
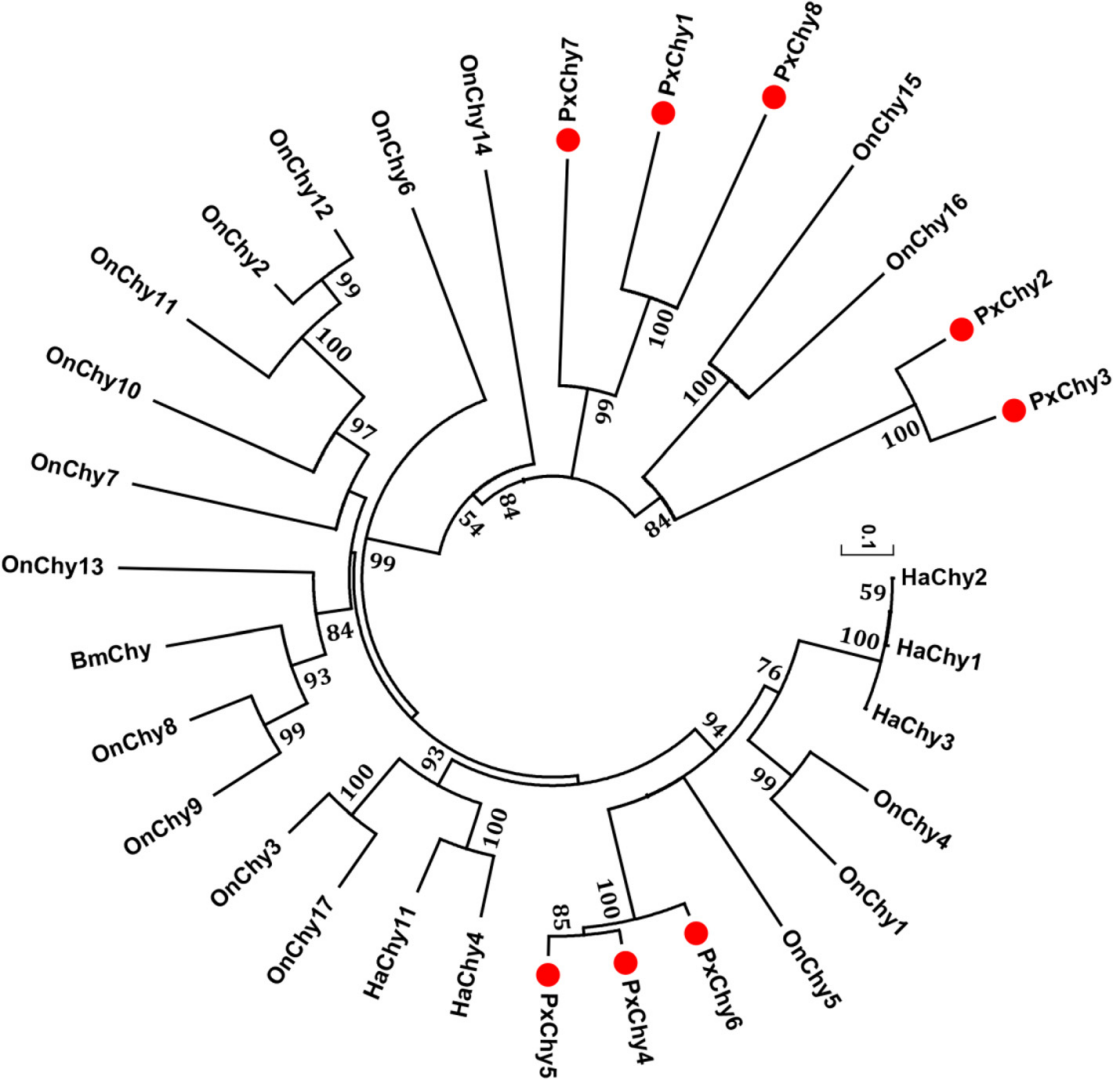
Phylogenetic relationship of chymotrypsins in *P. xylostella* and other Lepidoptera species. The phylogenetic tree was constructed using MEGA 6.06 with neighbor joining approach based on Poisson model and pairwise deletion of gaps. The percentage bootstrap scores higher than 50 % are indicated on the nodes. The first two letters in each of the gene names represent the acronym of scientific name for a given species (Px: *Plutella xylostella*; On*: Ostrinia nubilalis*; Ha*: Helicoverpa armigera*; Bm: *Bombyx mori*). *P. xylostella* chymotrypsins are marked with red dots

Phylogenetic analysis of chymotrypsin genes showed that PxChy2 and PxChy3 were grouped with OnChy15 and OnChy16 (Fig. 3). In *O. nubilalis*, the expression of OnChy15 is too low to be detected by RT-PCR, but OnChy16 is expressed in the foregut and midgut [29]. PxChys 4–6 were clustered with the genes in *O. nubilalis* (OnChys 1, 4, and 5) and *H. armigera* (HaChys 1–3), with a relatively high level of similarity (51.7 %) among them. OnChy1 and OnChy4 are expressed only in the foregut and midgut, while OnChy5 is expressed in all three gut sections (foregut, midgut and hindgut). OnChy5 is significantly up-regulated after a 24-h exposure to Cry1Ab, suggesting that it may be conducive to the degradation of activated Cry1Ab toxin in *O. nubilalis* [29].

### Clip-domain SP/SPH genes

In this study, 13 PxCLIPs were predicted in *P. xylostella* (Additional file 1: Table S1), which is close to the 14 serine proteases and their homologs linked to the clip-domain in *M. sexta* [40], 18 in *B. mori* [10] and *A. mellifera* [5], but fewer than the 37 in *D. melanogaster* [8] and 41 in *A. gambiae* [19]. Among the 13 PxCLIPs, PxCLIP11 contained two clip domains, for a total of 14 clip domains detected, suggesting its potential function as a proPO-activating enzyme as reported in the other Lepidoptera such as *M. sexta* [41, 42] and *B. mori* [43].

The number of residues between Cys-1 and Cys-6 varied from 43 to 53 (Additional file 6: Figure S4), which was consistent with the range of clip domains previously documented in insects, ranging from 37 to 55 residues [9]. The number of residues between Cys-1 and Cys-2 varied from two to ten, but was constant between Cys-2 and Cys-3 with five residues in all the clip domains (Additional file 6: Figure S4). Clip domains are usually divided into two groups depending on the number of residues between Cys-3 and Cys-4, with group 1 clip domain having 8-17 residues and group 2 having 22–26 residues [9]. Five clip domains in PxCLIPs 1, 7, 9, 10, and 12 contained 14–17 residues between Cys-3 and Cys-4, indicating that they were of group 1, whereas the remaining clip domains had 22–25 residues in this region and were of group 2. Previous research indicates that terminal proteinases have either an Arg or Lys residue at their activation sites that are replaced by Leu, His or Ser in penultimate proteinases [44]. The predicted proteolytic activation sites of 13 PxCLIPs showed that PxCLIPs 2, 4–9, 11, and 13 either had an Arg or Lys residue but PxCLIP1 had another residue at its activation site (Additional file 1: Table S1). We therefore propose that PxCLIPs 2, 4–9, 11, and 13 are terminal proteinases in the cascade pathway, while the PxCLIP1 may belong to penultimate proteinases.

**Fig. 4:**
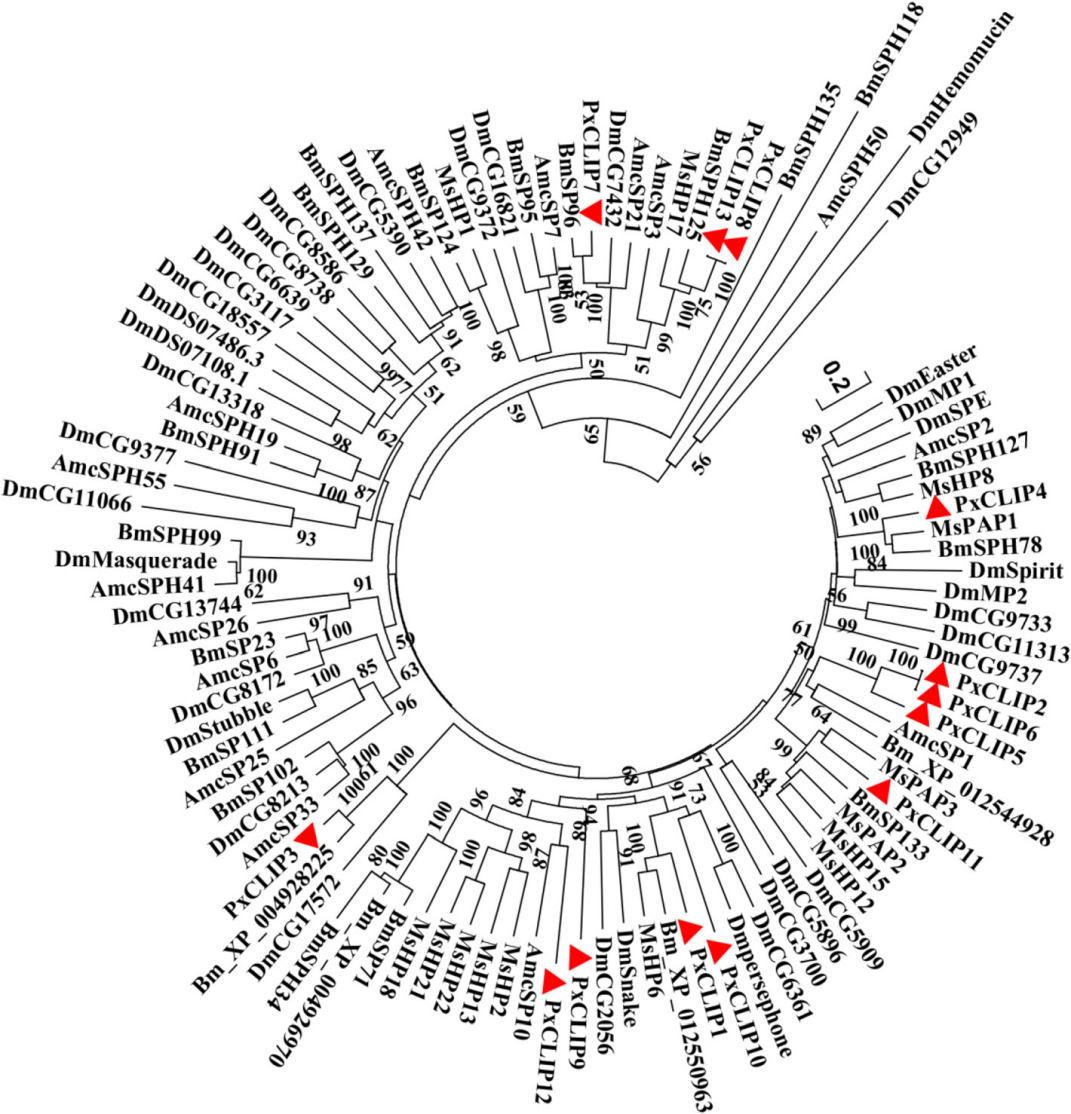
Phylogenetic relationship of CLIPs in *P. xylostella* and other four insect species. The phylogenetic tree was constructed using MEGA 6.06 with neighbor joining approach based on Poisson model and pairwise deletion of gaps. The percentage bootstrap scores higher than 50 % are indicated on the nodes. The first two letters in each of the gene names represent the acronym of scientific name for a given species (Dm: *Drosophila melanogaster*; Bm: *Bombyx mori*; Ms: *Manduca sexta*; Am: *Apis mellifica*). *P. xylostella* CLIPs are marked with red triangles

The phylogenetic tree including *P. xylostella*, *B. mori*, *M. sexta*, *D. melanogaster*, and *A. mellifera* clip-domain SPs/SPHs showed that seven of 13 PxCLIPs were clustered together with those of two Lepidoptera species *B. mori* and *M. sexta* (Fig. 4). For instance, PxCLIP11 was grouped with MsPAP3, and PxCLIP4 was homologous to MsPAP1 and BmSPH78. In *M. sexta*, PAP1 (proPO-activating proteinase 1) and PAP3 (proPO-activating proteinase 3), containing a group 2 clip domain, are terminal proteinases in the cascade pathway and known to be involved in proPO cleavage and activation [42, 45]. In *B. mori*, BmSPH78 contains a group 2 clip domain and is markedly up-regulated after induction, suggesting that it may have a similar function to its tobacco hornworm homolog MsPAP1 [10]. PxCLIP4 and PxCLIP11 consisted of a group 2 clip domain and were also terminal proteinases, which have been reported and named PxPAPa (JQ581597) and PxPAPb (JQ581598) [26]. MsHP6, a hemolymph proteinase of *M. sexta* with a group 1 clip domain, is a penultimate proteinase in two different immune pathways, leading to activation of proPO and the melanization response, and activation of hemolymph proteinase 8 (HP8), which stimulates a Toll-like pathway [44]. PxCLIP1 was considered homologous to Bm_XP_012550963 and MsHP6, implying that PxCLIP1 could have similar biological functions. PxCLIP8 and PxCLIP13 were clustered with BmSPH125, MsHP17 and AmcSP3 (a clip domain serine protease in *A. mellifera*), with a high level of similarity (75.5 %) among them. BmSPH125 contains a group 2 clip domain, which has been suggested to participate in pathogenic microorganism resistance in *B. mori* [10]. In *M. sexta*, the expression of MsHP17 (hemolymph proteinase 17) is not detectable in hemocytes or fat body, however, it is produced in both tissues after the microbial infection [40]. PxCLIP3 was clustered with Bm_XP_004928225 and DmCG17572 (a clip domain serine protease homolog in *D. melanogaster*), but the functions of these two clip serine proteases were unclear. However, PxCLIP10 was grouped with DmPersephone and DmCG6361. In *D. melanogaster*, Persephone serves in the Toll pathway, which can be activated by fungal and bacterial proteinases [46]. DmCG6361 is involved in systemic wound response, which is required for host protection against wounds and upregulated in response to septic infection in a Toll- and IMD-dependent manner [47].

### Other SP/SPH genes

Previous research has indicated that Nudel and gastrulation defective (Gd) are the key components of dorso-ventral axis establishment in the *Drosophila* embryo [48]. The Toll-Dorsal pathway is also conducive to other processes including immunity, morphogenetic movements and muscle development at later developmental stages [48]. Stubble has a type II transmembrane domain, which is indispensable for leg and wing morphogenesis [49]. Nudel, Gd and stubble genes from *D. melanogaster* were used to search for the same sequences in the *P. xylostella* genome, and one Nudel, one Gd and eight stubble-like genes were predicted in *P. xylostella* (Additional file 1: Table S1).

**Fig. 5:**
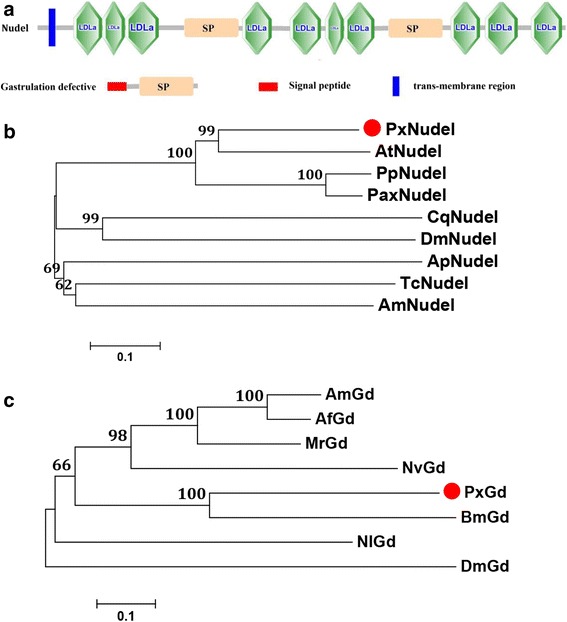
Domain architecture (**a**) and phylogenetic analysis of Nudel (**b**) and gastrulation defective (Gd) (**c**) using MEGA 6.06 with neighbor joining approach based on Poisson model and pairwise deletion of gaps. The sequences used for phylogenetic analysis of the Nudel gene in *P. xylostella* and other seven insect species were obtained from GenBank (NCBI) with accession numbers: Px003732 (PxNudel, *Plutella xylostella*), XP_001944581 (ApNudel, *Acyrthosiphon pisum*), XP_006559739 (AmNudel, *Apis mellifica*), NP_523947 (DmNudel, *Drosophila melanogaster*), XP_013138700 (PpNudel, *Papilio polytes*), XP_013190169 (AtNudel, *Amyelois transitella*), XP_001843380 (CqNudel, *Culex quinquefasciatus*) and XP_008201274 (TcNudel, *Tribolium castaneum*). The sequences used for phylogenetic analysis of the Gd gene in *P. xylostella* and other seven insect species were obtained from GenBank (NCBI) with accession numbers: Px006975 (PxGd, *Plutella xylostella*), KJ512078 (NlGd, *Nilaparvata lugens*), XP_003690498 (AfGd, *Apis florea*), XP_006563318 (AmGd, *Apis mellifica*), XP_003704669 (MrGd, *Megachile rotundata*), XP_003427708 (NvGd, *Nasonia vitripennis*), XP_004929031 (BmGd, *Bombyx mori*) and NP_511134 (DmGd, *Drosophila melanogaster*). *P. xylostella* Nudel and Gd are marked with red dots

The PxNudel gene (Px003732) was composed of a similar structure to DmNudel, including a transmembrane region, eight intact LDLA repeats and two Tryp_SPc domains (Fig. 5a). Multiple sequence alignment showed that *P. xylostella* Nudel and other Lepidoptera Nudels contained eight LDLA domains, three conserved motifs and putative activation residues (Additional file 7: Figure S5), with a 42 % level of identity among them. Phylogenetic analysis indicated that PxNudel was more closely clustered with Lepidoptera Nudels (AtNudel, PpNudel, PpNudel and PaxNudel) and Diptera Nudels (CqNudel and DmNudel), rather than those in the other three insect species (ApNudel, TcNudel and AmNudel) (Fig. 5b). The PxGd gene (Px006975) encompassed both a signal peptide and a Tryp_SPc domain (Fig. 5a), which was most closely related to the counterpart of *B. mori* (Fig. 5c). However,multiple sequence alignments showed a low sequence similarity with the Gds of other species (Additional file 8: Figure S6). Future experiments are needed to test whether PxGd is involved in early embryonic development. *P. xylostella* stubble-like proteins lacked the transmembrane domains found in the *D. melanogaster* stubble, but phylogenetic analysis showed that these genes were homologous to those of *B. mori*, *Musca domestica*, *A. pisum* and *Bombus terrestris* (Additional file 9: Figure S7). Eight stubble-like genes in *P. xylostella* were predicted, while five were identified in *N. lugens* and only one in *D. melanogaster*, implying that the abundant stubble-like genes might be involved in some physiological processes.

### Expression profiling of the PxSPs and PxSPHs

#### Stage-specific expression profiling

**Fig. 6:**
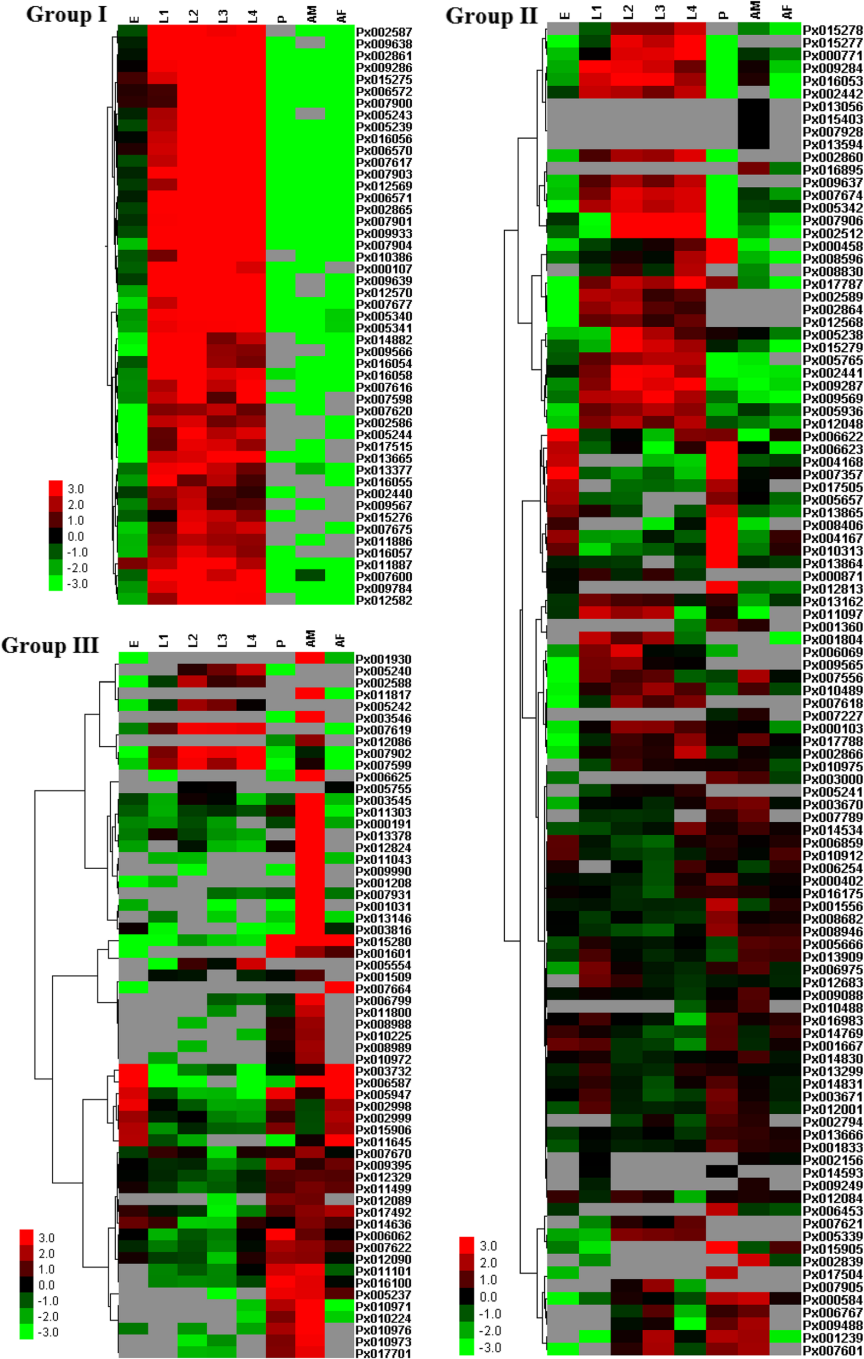
Expression profiling of the *P. xylostella* SP and SPH genes across different developmental stages. The log_2_ RPKM values are presented by bar colors where the darker red represents higher expression values, the darker green represents lower expression values, and the gray represents missing values. E, eggs; L1, 1st-instar larvae; L2, 2nd-instar larvae; L3, 3rd-instar larvae; L4, 4th-instar larvae; P, pupae; AM, adult males; AF, adult females. The RPKM values are given in the Additional file 10: Table S3

Expression of the 221 PxSP and PxSPH genes was profiled using RNA-seq data from the different developmental stages of the insecticide-susceptible strain (Fuzhou-S) including eggs, larvae, pupae and adults. The hierarchical clustering was used to describe the various relative levels of expression of SP and SPH genes, which could be differentiated into three distinct groups (Fig. 6). Group I included genes that had higher expression in larval stages than any other life stages. The larva is a crucial assimilatory stage in the life history of insects [50], especially holometabolous form such as *P. xylostella*, and serine protease genes have been identified from the larva of several insect species and presumed to function in protein digestion [2, 51]. The genes in Group II displayed differential expressions, indicating that they may play diverse physiological roles in *P. xylostella*. For example, 32 genes tended to have higher expression in larval stages than in other stages, suggesting their potential roles in digestion. Nine genes were highly expressed in eggs and pupae. Px001833 was expressed in pupae and adults, and Px012001 showed a high expression in the first instar larvae, pupae and adult males. Px001833 and Px012001 were homologous to MsPAP1 and MsPAP3, respectively, playing roles in proPO cleavage and activation [42, 45].

Group III consisted of 62 genes, six of which were expressed with moderate levels in a given larval stage, but with undetectable or very low levels in eggs, pupae and adults. Seventeen of the 62 genes showed exclusive and high expressions in adults, and exhibited a sex-specific pattern with 16 genes highly expressed in males and one gene highly expressed in females. Four genes had high levels of expression in eggs, pupae and adult females, and nine genes were highly expressed in pupae and adult males. Px003732 (PxNudel) was expressed highly in eggs and adult females. In the *Drosophila* embryo, this gene functions as a key component of dorso-ventral axis establishment [48]. The qPCR analysis confirmed stage-specific expression patterns of the ten genes that displayed high expressions based on RNA-seq data (Additional file 11: Figure S8). The differential expression patterns of serine proteases and homologs in different stages suggested their functional diversity in *P. xylostella*, but their functions remain to be identified.

#### Tissue-specific expression profiling

RNA-seq analysis showed that 196 of the 221 PxSP and PxSPH genes exhibited expressions in at least one tissue (Additional file 12: Figure S9). The midgut of 4th-instar larvae had the highest number (161) of genes expressed among the four tissues, while the number of genes expressed in the heads of 4th-instar larvae, adult males and females was 148, 130 and 140, respectively. Further analysis revealed that most of the PxSP and PxSPH genes showed high levels of expression in the midgut of the 4th-instar larvae, but tended to express at very low levels in the head. Previous research has demonstrated that the midgut is the most important tissue as an organ of food digestion and nutrient absorption [25]. Other work has suggested that the rich larval midgut-specific serine proteinases reduce the adverse effects of plant protease inhibitors through differential expressions in response to feeding on different plant hosts [27, 52].

## Conclusions

In this study, we identified and characterized 221 putative SP/SPH members in *P. xylostella*, along with their expression profiles in different developmental stages and tissues. Our results reveal that the SP/SPH complex may play various functions in *P. xylostella*, especially in digestion as suggested by highly expressed genes in the midgut and larval stages, and possibly in the immune system based on phylogenetic analysis of the CLIP genes. However, their functions remain to be validated by further molecular studies, such as gene cloning and protein identification, RNAi, and/or CRISPR-Cas9 to define target genes that may help explain this pest’s biological success, especially its capacity to adapt rapidly to the toxins in insecticides and occurring naturally in host plants. Such work offers scope to generate additional genetic and metabolic targets for pest management in the future.

## Methods

### Identification and characterization of the PxSPs and PxSPHs

The SP and SPH sequences from *B. mori*, *D. melanogaster*, *T. castaneum*, *A. mellifera*, *O. nubilalis, H. armigera* and *N. lugens* were downloaded from NCBI GenBank (http://www.ncbi.nlm.nih.gov/genbank/). They were queried against the DBM database (http://iae.fafu.edu.cn/DBM/) using the BLASTP program with an E value < 10^-7^. The predicted genes were then manually checked using NCBI and UniPort online BLASTP, with threshold of E-value < 10^-25^. The complete open read frames (ORFs) of SP/SPH genes were predicted with the method used by Yu et al. [53].

The predicted sequences were divided into serine proteases (SPs) and serine protease homologs (SPHs) based on the conserved catalytic triad residues, including His (H), Asp (D) and Ser (S). Sequence with the TAAHC, DIAL, and GDSGGP domains was considered to be SP, otherwise, sequence was taken to be SPH [5, 8]. The clans of putative sequences were determined using the MEROPS online service [54]. Signal peptides were analyzed using SignalP4.0 (http://www.cbs.dtu.dk/services/SignalP/).

The putative amino acid sequence of each SP and SPH was predicted for various domains and motifs by PROSITE (http://us.expasy.org/prosite), CDART (http://www.ncbi.nlm.nih.gov/Structure/lexington/lexington.cgi) and SMART (http://smart.embl-heidelberg.de). Some SPs and SPHs containing clip domain(s) were designated as PxCLIPs.

Residues 189, 216 and 226 determined the primary substrate-binding pocket based on the sequence alignment. SP consisting of Asp189, Gly216 and Gly/Ala/Ser226 was predicted to be trypsin; whereas SP with position 189 being replaced by Ser/Thr/Gly was presumed to be chymotrypsin [7, 8].

### Gene localization on scaffolds

The information of PxSP and PxSPH genes regarding their loci and orientation was obtained from the DBM genome (http://iae.fafu.edu.cn/DBM/). These genes were mapped on scaffolds using Mapchart with default parameters [55], and illustrated in Fig. 1a and Additional file 3: Figure S1. Tandem duplication is defined as neighboring homologous genes on single scaffold with ≤ five genes between them [53, 56, 57].

The gene structure information was obtained from the DBM genome (http://iae.fafu.edu.cn/DBM/) and exon-intron structures and intron phases of some trypsin genes were drawn by the web server Gene Structure Display Server (GSDS: http://gsds.cbi.pku.edu.cn).

### Sequence alignment and phylogenetic analysis

The functional serine protease domains of the *P. xylostella* SPs and SPHs were aligned with the best-matched homologs of other insect species by the ClustalX version 2.0 [58]. A phylogenetic tree was constructed with MEGA 6.06 [59] using the neighbor-joining (NJ) method by Poisson model with a bootstrap value of 1000 replicates.

### Ethics statement

*P. xylostella* is not protected under any legislation in China, as a protected or endangered species regulating or restricting its collection. No specific permits were required for collecting the larvae from the field nor was animal ethics approval required for work with this invertebrate.

### Insect strain and rearing

A susceptible strain (Fuzhou-S) of *P. xylostella* was originally obtained from cruciferous vegetable fields in Fuzhou in 2004 and used for genome sequencing [27]. The population was maintained on radish seedlings at 25 ± 2 °C, 70 ~ 80 % RH, 16 : 8 h = light : dark cycle. Adults were provided with cotton balls soaked in 10 ~ 20 % (v/v) honey solution as food. Newly laid eggs, 1st-, 2nd-, 3rd- and 4th-instar larvae, pupae and adults (regardless of gender) were collected and stored at -80 °C.

### Expression profiling of the PxSP and PxSPH genes

Based on the RNA-seq data previously completed in our laboratory, the expression patterns of the 221 PxSP and PxSPH genes were profiled using Cluster 3.0 software and visualized by Java TreeView [60]. The RPKM values were log_2_ transformed, and the clustered genes were illustrated in terms of their expression patterns by the similarity metric of Euclidean distance and clustering method of complete linkage [53]. The samples used in this study included eggs, 1st-, 2nd-, 3rd- and 4th-instar larvae, pupae, adults, midguts and heads of 4th-instar larvae, heads of adult males and adult females. The RPKM values were also given in Additional file 10: Tables S3 and Additional file 13 Table S4.

The total RNA of each sample was extracted using TRIzol (Invitrogen, USA) and digested with 1 μL gDNA Eraser (Takara Biotechnology (Japan) Co., Ltd.) for 2 min at 42 °C to remove contaminating genomic DNA. The template (cDNA) for qPCR was synthesized by total RNA (1 μg) using PrimeScript™ RT reagent Kit (TaKaRa, Japan) according to the manufacturer’s instructions. qPCR was performed in a total reaction volume of 20 μL, containing 10 μL of 2 × real-time PCR Mix (containing SYBR Green I), 0.4 μL of each primer (10 mmol/L), 2 μL of cDNA template from the relative samples (100 ng/μL final concentration), and 7.2 μL water in CFX96 Touch™ Real-Time PCR Detection System (Bio-Rad, USA). Following manufacturer’s instructions for the GoTaq qPCR Master Mix (Promega, USA), PCR was conducted with an initial denaturation at 95 °C for 3 min, followed by 40 cycles at 95 °C for 10 s and 60 °C for 30 s, and a final melt curve starting at 63 °C for 5 s up to 95 °C with 0.5 °C increments. Ten SP/SPH genes highly expressed in larval stages were selected for further validation of expression by qPCR and the primers were designed for qPCR performance (Additional file 14: Table S5). The *P. xylostella* ribosomal protein gene L32 was used as the housekeeping reference (forward primer: 5′-AAT CAG GCC AAT TTA CCG C-3′; reverse primer: 5′-CTG GGT TTA CGC CAG TTA CG-3′). Relative gene expression data were normalized against Ct values for the housekeeping gene.

The qPCR data were statistically analyzed using the R statistical program version 3.0.2, with the supplemented package ‘agricolae’ [61]. If the data satisfied normality assumption, one-way ANOVA was performed, otherwise the Kruskal-Wallis nonparametric test was used.

## Additional files

Additional file 1: Table S1. Description of serine protease (SP) and serine protease homolog (SPH) genes in P. xylostella. (XLSX 38 kb) Additional file 2: Table S2. The predicted amino acid sequences of 221 SPs/SPHs. (XLSX 55 kb) Additional file 3: Figure S1. Scaffold localization of PxSPs and PxSPHs in P. xylostella. (DOC 533 kb) Additional file 4: Figure S2. Multiple alignment of 38 P. xylostella trypsin genes along with well annotated trypsins: Aedes aegypti trypsin 3A1 (AaTry3A1), Anopheles gambiae trypsin-6 (AgTry6), Culex quinquefasciatus trypsin1 (CqTry1) and trypsin5 (CqTry5). (DOC 1860 kb) Additional file 5: Figure S3. Multiple alignment of 8 P. xylostella chymotrypsin genes along with HaChys 1-3 and OnChys 1-3. (DOC 1012 kb) Additional file 6: Figure S4. Alignment of 14 P. xylostella clip domain sequences along with the clip domains from BmSPH78, BmSPH125, MsHP6, MsHP8, MsPAP1 and MsPAP3 by Clustal X2. (PxCLIP11 has two clip domains represented by PxCLIP11a and PxCLIP11b). (DOC 352 kb) Additional file 7: Figure S5. Multiple alignment of P. xylostella Nudel gene along with other three Lepidoptera Nudels: Papilio Xuthus Nudel, PaxNudel (XP_013165117.1), Papilio polytes Nudel, PpNudel (XP_013138700.1) and Amyelois transitella Nudel, AtNudel (XP_013190169.1) by Clustal X2. (DOC 2247 kb) Additional file 8: Figure S6. Multiple alignment of P. xylostella Gd gene along with other insect species Gds, Nilaparvata lugens Gd, NlGd (AID60301.1); Apis mellifera Gd, AmGd (XP_006563318.1); Bombyx mori Gd, BmGd (XP_012548092.1); Drosophila melanogaster Gd, DmGd (ABG02140.1); Apis florea Gd, AfGd (XP_003690498.1); Nasonia vitripennis Gd, NvGd (XP_003427708.1), and Megachile rotundata Gd, MrGd (XP_012143735.1) by Clustal X2. (DOC 1166 kb) Additional file 9: Figure S7. Phylogenetic tree/analysis of the stubble genes in P. xylostella and other five insect species. (DOC 98 kb) Additional file 10: Table S3. RPKM values of the PxSP and PxSPH genes at different developmental stages obtained from the RNA-seq data. (XLSX 33 kb) Additional file 11: Figure S8. qPCR-based expression profiling of the SP and SPH genes across different developmental stages. (DOC 232 kb) Additional file 12: Figure S9. Illustration for expression profiling of the P. xylostella SP and SPH genes in different tissues, showing the hierarchical clustered groups of expression pattern. (DOC 142 kb) Additional file 13: Table S4. RPKM values of the PxSP and PxSPH genes at different developmental tissues obtained from the RNA-seq data. (XLSX 23 kb) Additional file 14: Table S5. Primers used for qPCR study on gene expression. (XLSX 11 kb)

## Abbreviations

Aa: *Aedes aegypti*
AaTry3A1: *A. aegypti* trypsin 3A1
AgTry6: *Anopheles gambie* trypsin-6
Am: *Apis mellifera*
AmcSP3: a clip domain serine protease in *A. mellifera*
Bm: *Bombyx mori*
Bt: *Bacillus thuringiensis*
Cq: *Culex quinquefasciatus*
CqTry1: *C. quinquefasciatus* trypsin1
Dm: *Drosophila melanogaster*
FRI: frizzled
Ha: *Helicoverpa armigera*
HaChy: *H. armigera* chymotrypsin
HaTry: *H. armigera* trypsin
LDLA: low-density lipoprotein receptor class A
Ms: *Manduca sexta*
MsHP17: *M. sexta* hemolymph proteinase 17
MsHP6: *M. sexta* hemolymph proteinase 6
MsHP8: *M. sexta* hemolymph proteinase 8
MsPAP1: *M. sexta* proPO-activating proteinase 1
MsPAP3: *M. sexta* proPO-activating proteinase 3
On: *Ostrinia nubilalis*
OnChy: *O. nubilalis* chymotrypsin
OnTry: *O. nubilalis* trypsin
proPO: prophenoloxidase
Px: *Plutella xylostella*
PxChy: *P. xylostella* chymotrypsin
PxTry: *P. xylostella* trypsin
SPHs: serine protease homologs
SPs: serine proteases
SR: scavenger receptor Cys-rich
Tryp_SPc: an intact trypsin-like serine protease catalytic triad

## References

[1] Hedstrom L (2002). Serine protease mechanism and specificity. Chemical Rev.

[2] Li J, Choo YM, Lee KS, Je YH, Woo SD, Kim I (2005). A serine protease gene from the firefly, Pyrocoelia rufa: gene structure, expression, and enzyme activity. Biotechnol Lett.

[3] Choo YM, Lee KS, Yoon HJ, Lee SB, Kim JH, Sohn HD (2007). A serine protease from the midgut of the bumblebee, Bombus ignites (Hymenoptera: Apidae): cDNA cloning, gene structure, expression and enzyme activity. Eur J Entomol.

[4] Krem MM, Di Cera E (2002). Evolution of enzyme cascades from embryonic development to blood coagulation. Trends Biochem Sci.

[5] Zou Z, Lopez DL, Kanost MR, Evans JD, Jiang H (2006). Comparative analysis of serine protease-related genes in the honey bee genome: possible involvement in embryonic development and innate immunity. Insect Mol Biol.

[6] Jang IH, Nam HJ, Lee WJ (2008). CLIP-domain serine proteases in Drosophila innate immunity. BMB Rep.

[7] Perona JJ, Craik CS (1995). Structural basis of substrate specificity in the serine proteases. Protein Sci.

[8] Ross J, Jiang H, Kanost MR, Wang Y (2003). Serine proteases and their homologs in the Drosophila melanogaster genome: an initial analysis of sequence conservation and phylogenetic relationships. Gene.

[9] Jiang H, Kanost MR (2000). The clip-domain family of serine proteinases in arthropods. Insect Biochem Mol Biol.

[10] Zhao P, Wang G, Dong Z, Duan J, Xu P, Cheng T (2010). Genome-wide identification and expression analysis of serine proteases and homologs in the silkworm Bombyx mori. BMC Genomics.

[11] Bao YY, Qin X, Yu B, Chen LB, Wang ZC, Zhang CX (2014). Genomic insights into the serine protease gene family and expression profile analysis in the planthopper. Nilaparvata lugens BMC Genomics.

[12] Yu X, Jiang H, Wang Y, Kanost MR (2003). Nonproteolytic serine proteinase homologs are involved in prophenoloxidase activation in the tobacco hornworm, Manduca sexta. Insect Biochem Mol Biol.

[13] Gupta S, Wang Y, Jiang H (2005). Manduca sexta prophenoloxidase (proPO) activation requires proPO-activating proteinase (PAP) and serine proteinase homologs (SPHs) simultaneously. Insect Biochem Mol Biol.

[14] Felfoldi G, Eleftherianos I, Ffrench-Constant RH, Venekei I (2011). A serine proteinase homologue, SPH-3, plays a central role in insect immunity. J Immunol.

[15] Murugasu-Oei B, Rodrigues V, Yang X, Chia W (1995). Masquerade: a novel secreted serine protease-like molecule is required for somatic muscle attachment in the Drosophila embryo. Genes Dev.

[16] Povelones M, Bhagavatula L, Yassine H, Tan LA, Upton LM, Osta MA (2013). The CLIP-domain serine protease homolog SPCLIP1 regulates complement recruitment to microbial surfaces in the malaria mosquito Anopheles gambiae. PLoS Pathog.

[17] Huang T, Wang H, Lee SY, Johansson MW, Soderhall K, Cerenius L (2000). A cell adhesion protein from the crayfish Pacifastacus leniusculus, a serine proteinase homologue similar to Drosophila Masquerade. J Biol Chem.

[18] Zhang Q, Liu H, Chen R, Shen KL, Wang K (2013). Identification of a serine proteinase homolog (Sp-SPH) involved in immune defense in the mud crab Scylla paramamosain. PLoS One.

[19] Christophides GK, Zdobnov E, Barillas-Mury C, Birney E, Blandin S, Blass C (2002). Immunity-related genes and gene families in Anopheles gambiae. Science.

[20] Zou Z, Evans JD, Lu Z, Zhao P, Williams M, Sumathipala N (2007). Comparative genomic analysis of the Tribolium immune system. Genome Biol.

[21] Tanaka H, Ishibashi J, Fujita K, Nakajima Y, Sagisaka A, Tomimoto K (2008). A genome-wide analysis of genes and gene families involved in innate immunity of Bombyx mori. Insect Biochem Mol Biol.

[22] Zalucki MP, Shabbir A, Silva R, Adamson D, Liu SS, Furlong MJ (2012). Estimating the economic cost of one of the world’s major insect pests, *Plutella xylostella* (lepidoptera: plutellidae): just how long is a piece of string?. J Econ Entomol.

[23] Tabashnik BE, Huang F, Ghimire MN, Leonard BR, Siegfried BD, Rangasamy M (2011). Efficacy of genetically modified Bt toxins against insects with different genetic mechanisms of resistance. Nat Biotechnol.

[24] Xia X, Yu L, Xue M, Yu X, Vasseur L, Gurr GM (2015). Genome-wide characterization and expression profiling of immune genes in the diamondback moth, Plutella xylostella (L.). Sci Rep.

[25] Shi M, Zhu N, Yi Y, Chen XX (2013). Four serine protease cDNAs from the midgut of Plutella xylostella and their proteinase activity are influenced by the endoparasitoid, Cotesia vestalis. Arch Insect Biochem Physiol.

[26] Shi M, Chen XY, Zhu N, Chen XX (2014). Molecular identification of two prophenoloxidase-activating proteases from the hemocytes of Plutella xylostella (Lepidoptera: Plutellidae) and their transcript abundance changes in response to microbial challenges. J Insect Sci.

[27] You M, Yue Z, He W, Yang X, Yang G, Xie M (2013). A heterozygous moth genome provides insights into herbivory and detoxification. Nat Genet.

[28] Wolfson JL, Murdock LL (1990). Diversity in digestive proteinase activity among insects. J Chem Ecol.

[29] Yao J, Buschman LL, Oppert B, Khajuria C, Zhu K (2012). Characterization of cDNAs encoding serine proteases and their transcriptional responses to Cry1Ab protoxin in the gut of Ostrinia nubilalis larvae. PLoS One.

[30] Li H, Oppert B, Higgins RA, Huang F, Buschman LL, Gao J (2005). Characterization of cDNAs encoding three trypsin-like proteinases and mRNA quantitative analysis in Bt-resistant and -susceptible strains of Ostrinia nubilalis. Insect Biochem Mol Biol.

[31] Zhu-Salzman K, Zeng R (2015). Insect response to plant defensive protease inhibitors. Ann Rev Entomol.

[32] Bown DP, Wilkinson HS, Gatehouse JA (1997). Differentially regulated inhibitor-sensitive and insensitive proteinase genes from phytophagous insect pest Helicoverpa armigera, are members of complex multigene families. Insect Biochem Mol Biol.

[33] Zhu Y, Baker JE (1999). Characterization of midgut trypsin-like enzymes and three trypsinogen cDNAs from the lesser grain borer, Rhyzopertha dominica (Coleoptera: Bostrichidae). Insect Biochem Mol Biol.

[34] Muhlia-Almazán A, Sánchez-Paz A, García-Carreño FL (2008). Invertebrate trypsins: a review. J Comp Physiol B.

[35] Lehane SM, Assinder SJ, Lehane MJ (1998). Cloning, sequencing, temporal expression and tissue-specificity of two serine proteases from the midgut of the blood-feeding fly Stomoxys calcitrans. Eur J Biochem.

[36] Borges-Veloso A, Saboia-Vahia L, Dias-Lopes G, Domont GB, Britto C, Cuervo P (2015). In-depth characterization of trypsin-like serine peptidases in the midgut of the sugar fed Culex quinquefasciatus. Parasit Vectors.

[37] Wang S, Magoulas C, Hickey D (1999). Concerted evolution within a trypsin gene cluster in Drosophila. Mol Biol Evol.

[38] Chikate YR, Tamhane VA, Joshi RS, Gupta VS, Giri AP (2013). Differential protease activity augments polyphagy in Helicoverpa armigera. Insect Mol Biol.

[39] Yao J, Buschman LL, Lu N, Khajuria C, Zhu KY (2014). Changes in gene expression in the larval gut of Ostrinia nubilalis in response to Bacillus thuringiensis Cry1Ab protoxin ingestion. Toxins.

[40] Jiang H, Wang Y, Gu Y, Guo X, Zou Z, Scholz F (2005). Molecular identification of a bevy of serine proteinases in Manduca sexta hemolymph. Insect Biochem Mol Biol.

[41] Jiang H, Wang Y, Yu X, Kanost MR (2003). Prophenoloxidase-activating Proteinase-2 from Hemolymph of Manduca sexta. J Biol Chem.

[42] Jiang H, Wang Y, Yu XQ, Zhu Y, Kanost MR (2003). Prophenoloxidase-activating proteinase-3 (PAP-3) from Manduca sexta hemolymph: a clip-domain serine proteinase regulated by serpin-1 J and serine proteinase homologs. Insect Biochem Mol Biol.

[43] Satoh D, Horii A, Ochiai M, Ashida M (1999). Prophenoloxidase-activating enzyme of the silkworm, Bombyx mori. Purification, characterization, and cDNA cloning. J Biol Chem.

[44] An C, Ishibashi J, Ragan EJ, Jiang H, Kanost MR (2009). Functions of Manduca sexta hemolymph proteinases HP6 and HP8 in two innate immune pathways. J Biol Chem.

[45] Zou Z, Wang Y, Jiang H (2005). Manduca sexta prophenoloxidase activating proteinase-1 (PAP-1) gene: organization, expression, and regulation by immune and hormonal signals. Insect Biochem Mol Biol.

[46] Ligoxygakis P, Pelte N, Hoffmann JA, Reichhart JM (2002). Activation of Drosophila Toll during fungal infection by a blood serine protease. Science.

[47] Nam HJ, Jang IH, You H, Lee KA, Lee WJ (2012). Genetic evidence of a redox-dependent systemic wound response via Hayan Protease-Phenoloxidase system in Drosophila. EMBO J.

[48] Belvin MP, Anderson KV (1996). A conserved signaling pathway: the Drosophila toll-dorsal pathway. Ann Rev Cell Dev Biol.

[49] Bayer CA, Halsell SR, Fristrom JW, Kiehart DP, von Kalm L (2003). Genetic interactions between the RhoA and Stubble-stubbloid loci suggest a role for a type II transmembrane serine protease in intracellular signaling during Drosophila imaginal disc morphogenesis. Genetics.

[50] Chougule NP, Giri AP, Sainani MN, Gupta VS (2005). Gene expression patterns of Helicoverpa armigera gut proteases. Insect Biochem Mol Biol.

[51] Zhu Y, Liu X, Maddur AA, Oppert B, Chen M (2005). Cloning and characterization of chymotrypsin- and trypsin-like cDNAs from the gut of the Hessian fly [Mayetiola destructor (Say)]. Insect Biochem Mol Biol.

[52] Henniges-Janssen K, Reineke A, Heckel DG, Groot AT (2011). Complex inheritance of larval adaptation in Plutella xylostella to a novel host plant. Heredity.

[53] Yu L, Tang W, He W, Ma X, Vasseur L, Baxter SW (2015). Characterization and expression of the cytochrome P450 gene family in diamondback moth, Plutella xylostella (L.). Sci Rep.

[54] Rawlings ND, Morton FR (2008). The MEROPS batch BLAST: a tool to detect peptidases and their non-peptidase homologues in a genome. Biochimie.

[55] Voorrips RE (2002). MapChart: software for the graphical presentation of linkage maps and QTLs. J Hered.

[56] Zhao H, Ma H, Yu L, Wang X, Zhao J (2012). Genome-wide survey and expression analysis of amino acid transporter gene family in rice (Oryza sativa L.). PLoS One.

[57] Zhang Y, Gao M, Singer SD, Fei Z, Wang H, Wang X (2012). Genome-wide identification and analysis of the TIFY gene family in grape. PLoS One.

[58] De Hoon MJ, Imoto S, Nolan J, Miyano S (2004). Open source clustering software. Bioinformatics.

[59] Tamura K, Stecher G, Peterson D, Filipski A, Kumar S (2013). MEGA6: Molecular Evolutionary Genetics Analysis version 6.0. Mol Biol Evol.

[60] Saldanha AJ (2004). Java Treeview--extensible visualization of microarray data. Bioinformatics.

[61] De Mendiburu F. Agricolae: statistical procedures for agricultural research. R package version 1.1–2. 2009; http://CRAN.R-project.org/package=agricolae.

