# Supplementary material for: Genome-wide identification and expression profiling of serine proteases and homologs in the diamondback moth, Plutella xylostella (L.)
Source: BMC Genomics. 2015 Dec 10;16:1054. doi: 10.1186/s12864-015-2243-4 (PMC4676143; doi:10.1186/s12864-015-2243-4)
Supplement: Additional file 3: Figure S1. — Scaffold localization of PxSPs and PxSPHs in P. xylostella. (DOC 533 kb) [file 12864_2015_2243_MOESM3_ESM.doc]

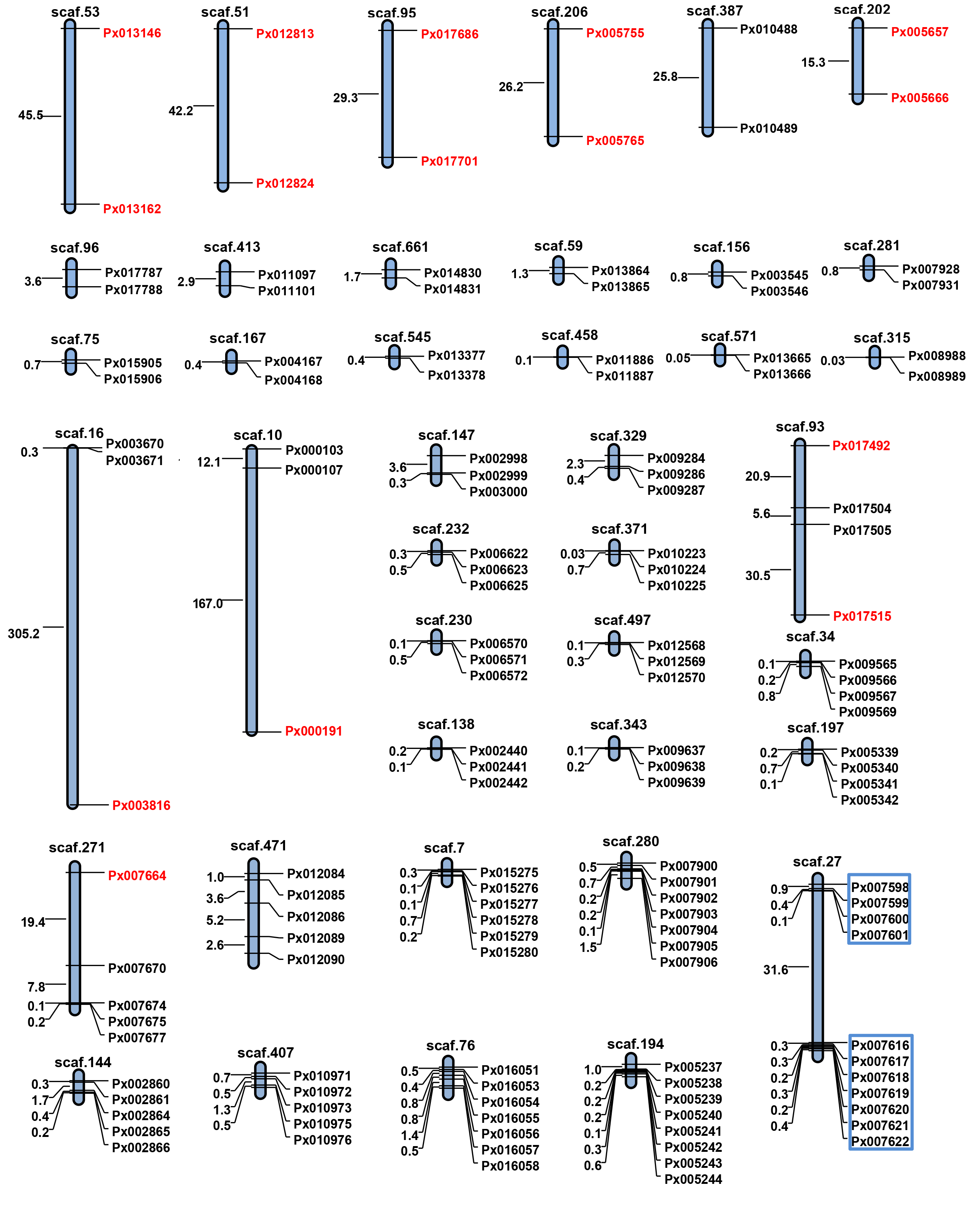
 **Additional file 3: Figure S1.** Scaffold localization of PxSPs and PxSPHs in *P. xylostella*. Clusters of the scaffolds containing ≥ 2 SP/SPH genes in *P.* *xylostella*, with 122 of the genes (in black) being predicted to be tandem duplication, genes that are not tandemly distributed are in red. Scaffold numbers are presented at the top of each bar. Gene IDs and the distance of two adjacent genes (10 kilobases) are showed on the right and left of the bars, respectively.
