# Supplementary material for: Genome-wide identification and expression profiling of serine proteases and homologs in the diamondback moth, Plutella xylostella (L.)
Source: BMC Genomics. 2015 Dec 10;16:1054. doi: 10.1186/s12864-015-2243-4 (PMC4676143; doi:10.1186/s12864-015-2243-4)
Supplement: Additional file 4: Figure S2. — Multiple alignment of 38 P. xylostella trypsin genes along with well annotated trypsins: Aedes aegypti trypsin 3A1 (AaTry3A1), Anopheles gambiae trypsin-6 (AgTry6), Culex quinquefasciatus trypsin1 (CqTry1) and trypsin5 (CqTry5). (DOC 1860 kb) [file 12864_2015_2243_MOESM4_ESM.doc]

**
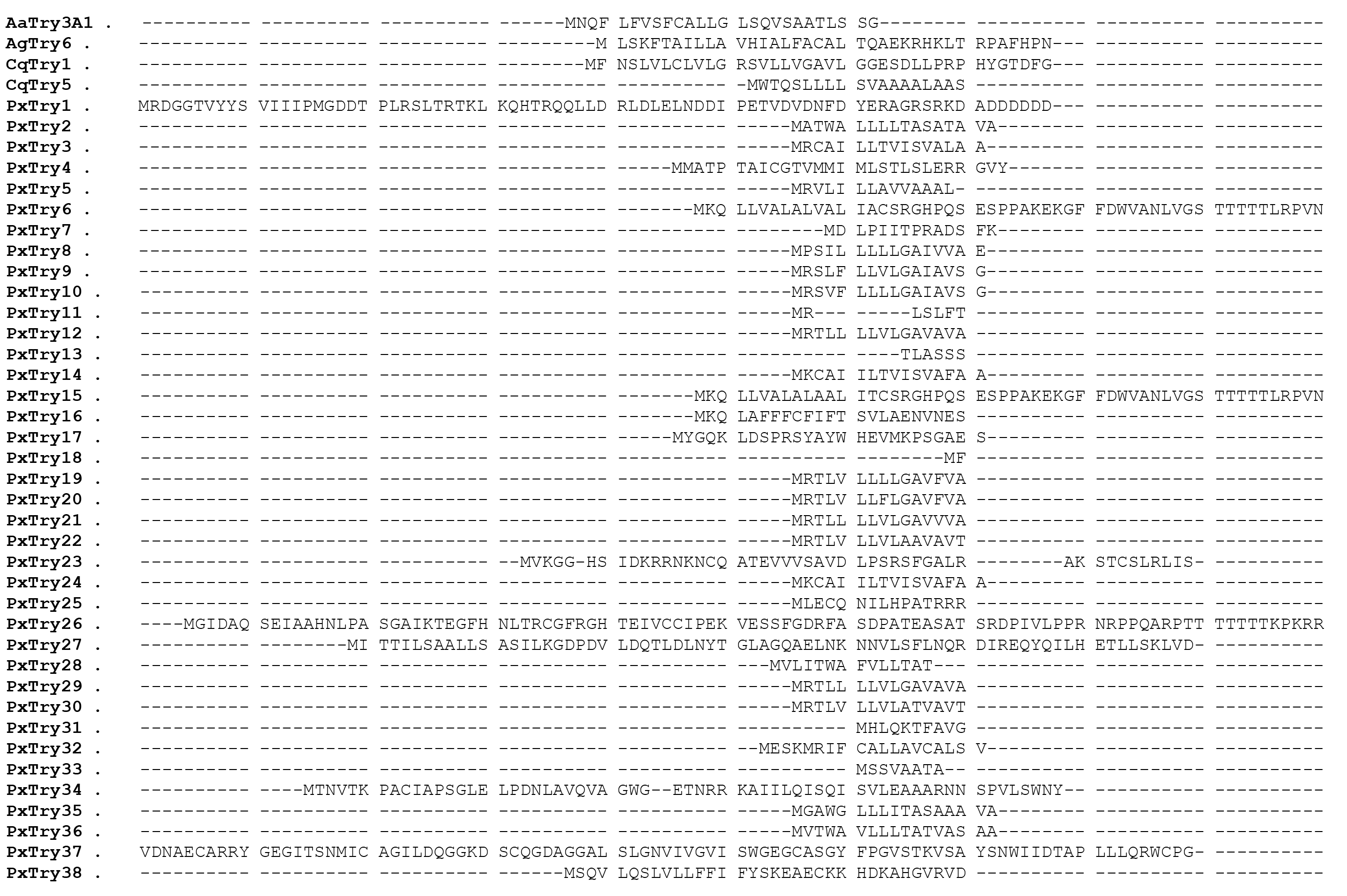
**

**
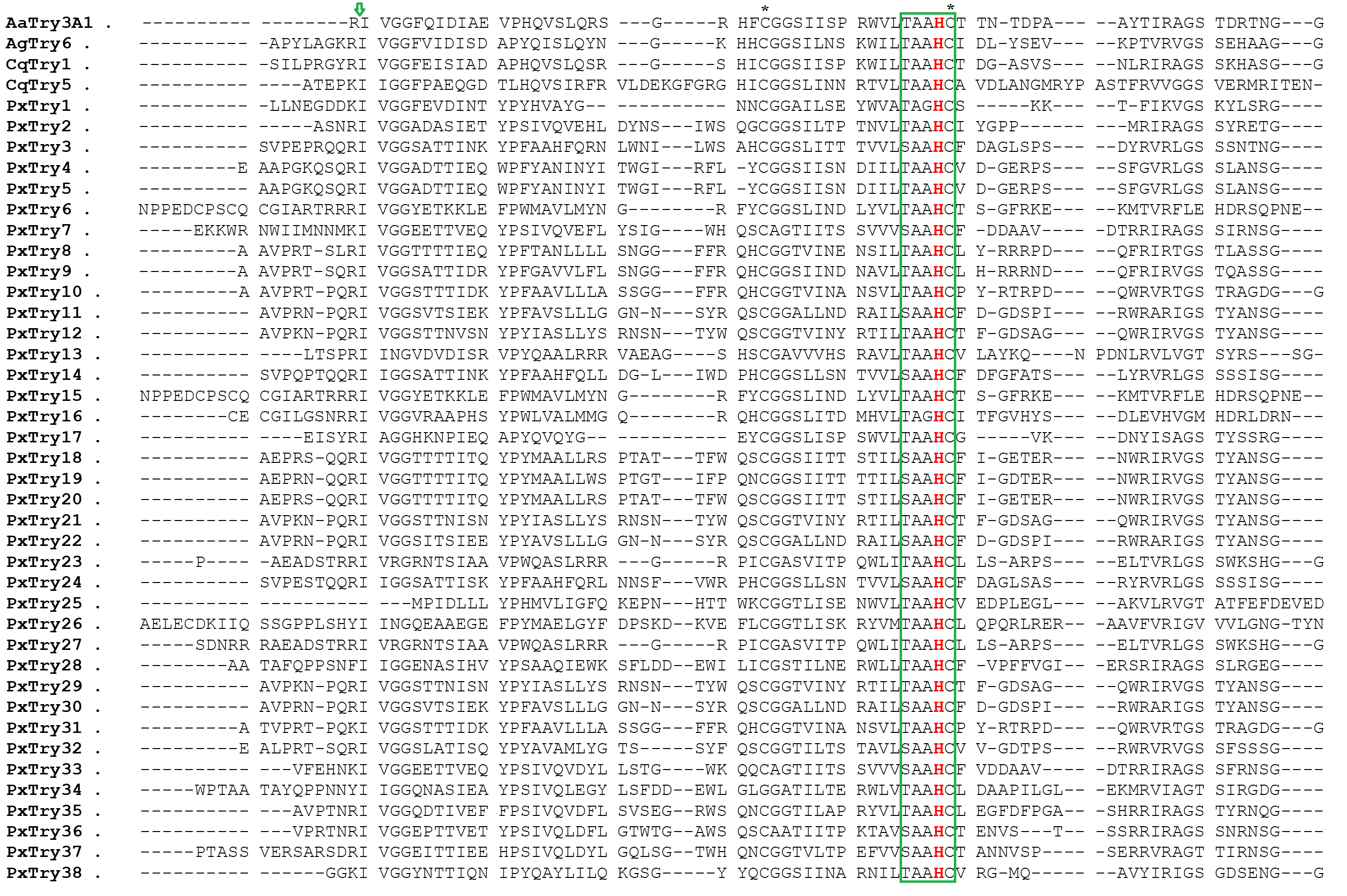
**

**
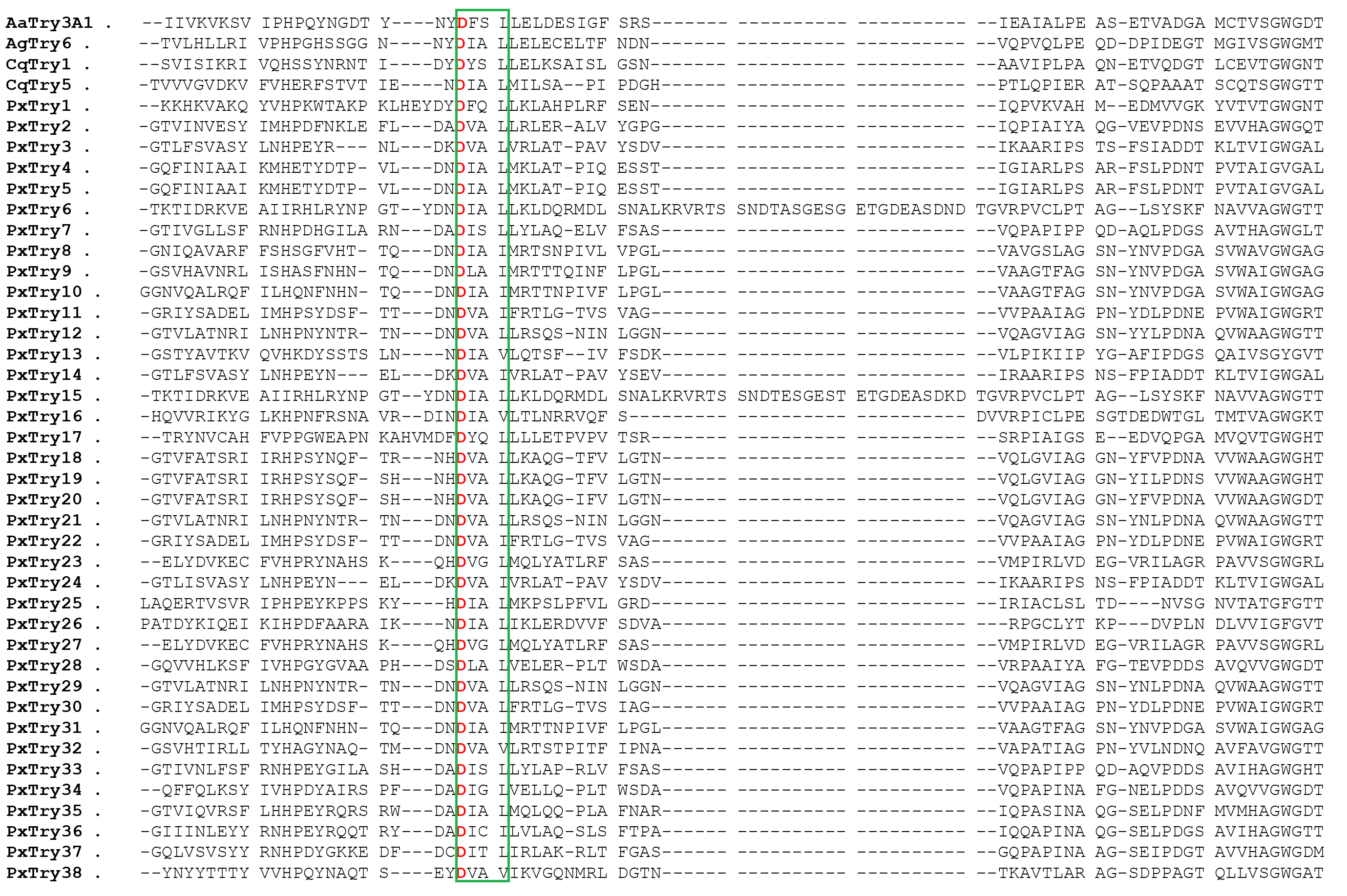
**

**
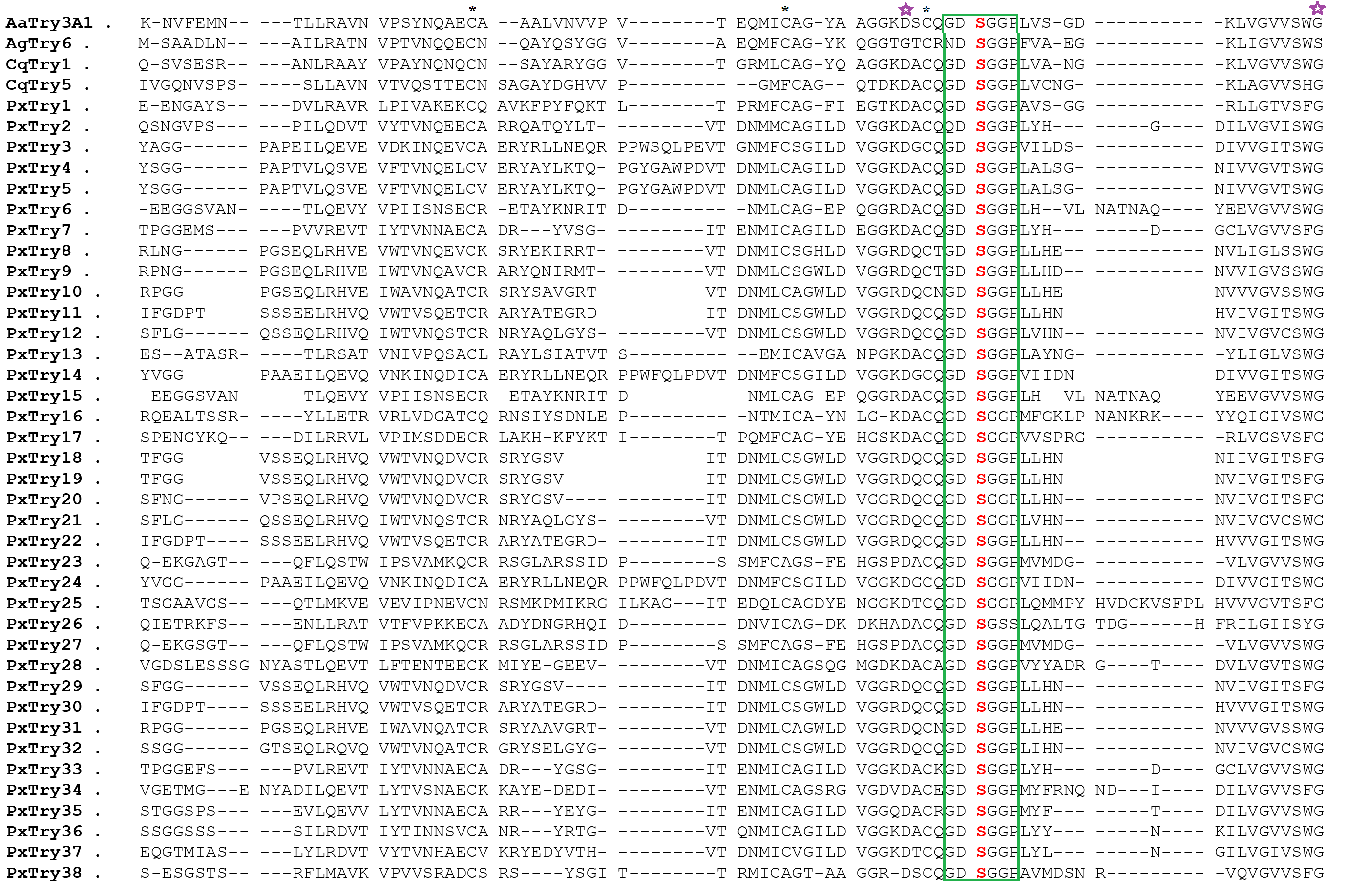
**

**
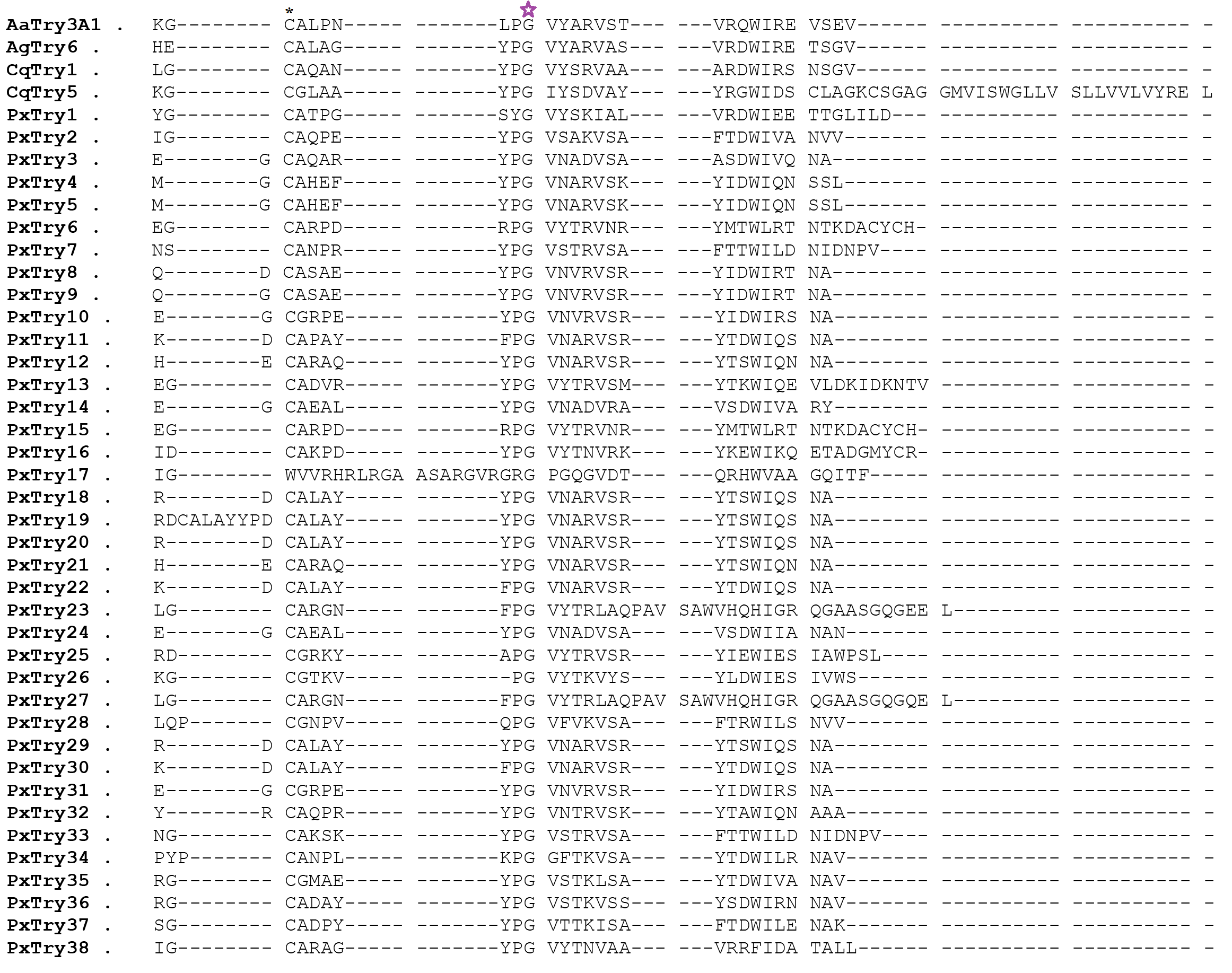
**

**Additional file 4: Figure S2.** Multiple alignment of 38 *P*. *xylostella* trypsin genes along with well annotated trypsins: *Aedes aegypti* trypsin 3A1 (AaTry3A1), *Anopheles gambiae* trypsin-6 (AgTry6), *Culex quinquefasciatus* trypsin1 (CqTry1) and trypsin5 (CqTry5). The catalytic triads (His, Asp and Ser) are in red, conserved regions (TAAHC, DIAL, and GDSGGP) are boxed in green, the putative autocatalytic site is marked with green arrow at the top, trypsin-determination residues are marked with purple star at the top; and the six cysteine residues are marked with black stars at the top.
