# Supplementary material for: Genome-wide identification and expression profiling of serine proteases and homologs in the diamondback moth, Plutella xylostella (L.)
Source: BMC Genomics. 2015 Dec 10;16:1054. doi: 10.1186/s12864-015-2243-4 (PMC4676143; doi:10.1186/s12864-015-2243-4)
Supplement: Additional file 5: Figure S3. — Multiple alignment of 8 P. xylostella chymotrypsin genes along with HaChys 1-3 and OnChys 1-3. (DOC 1012 kb) [file 12864_2015_2243_MOESM5_ESM.doc]

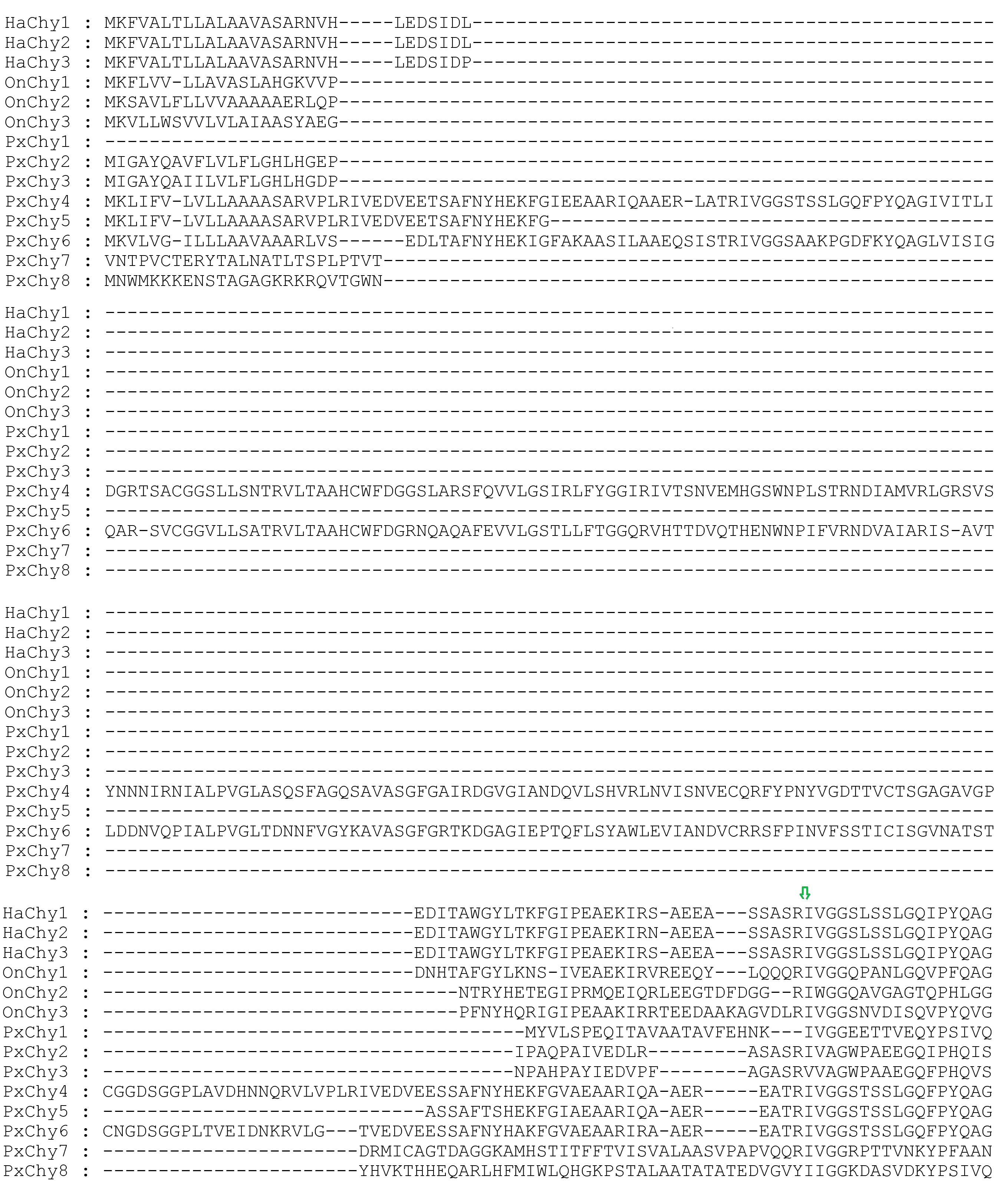


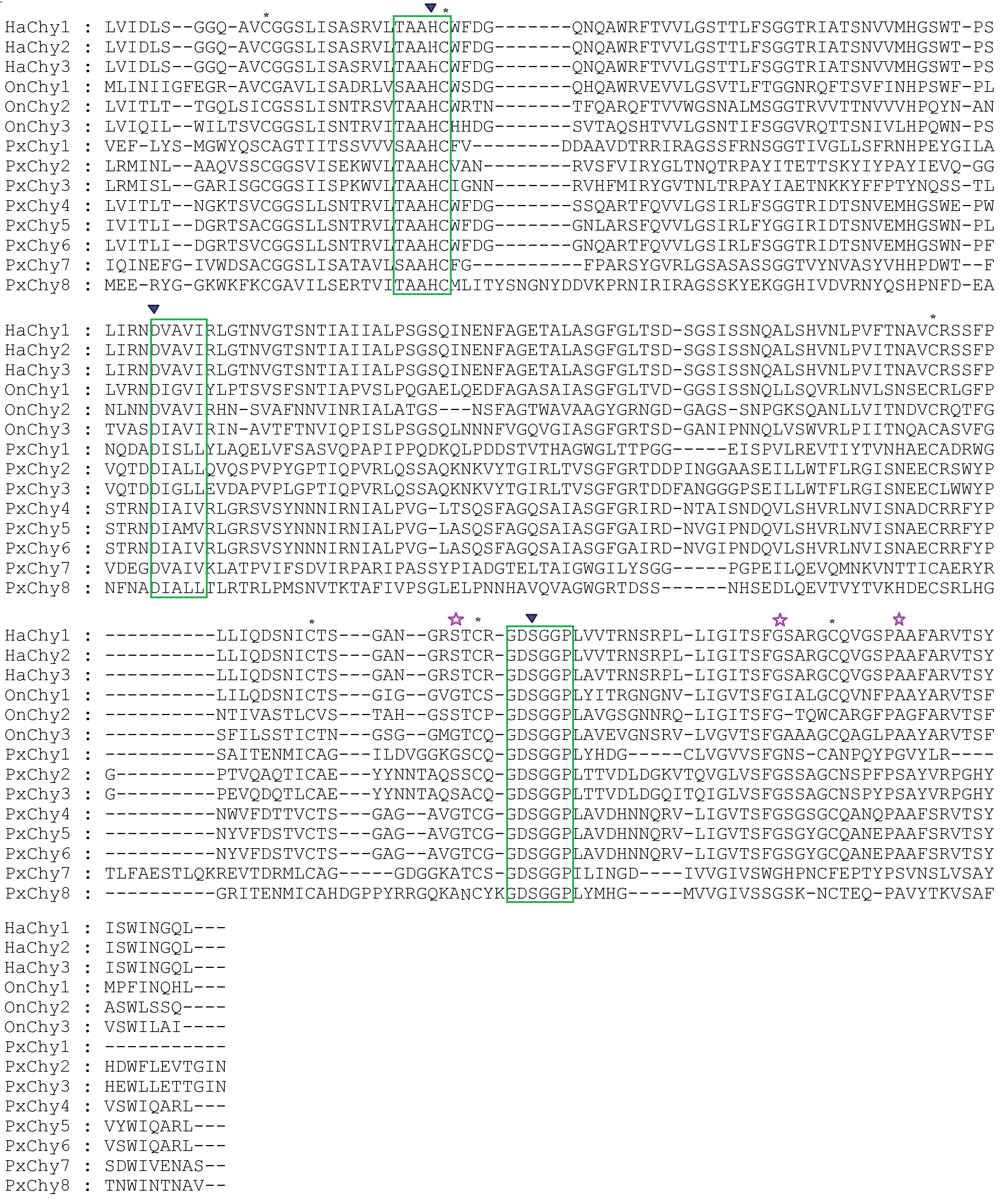


**Additional file 5: Figure S3.** Multiple alignment of 8 *P*. *xylostella* chymotrypsin genes along with HaChys 1-3 and OnChys 1-3. The catalytic triads (His, Asp and Ser) are marked with blue inverted triangle and conserved regions (TAAHC, DIAL, and GDSGGP) are boxed in green; the putative autocatalytic site is marked with a green arrow at the top; the chymotrypsin-determination residues are marked with purple star at the top; and the six cysteine residues are marked with black stars at the top.
