# Supplementary material for: Genome-wide identification and expression profiling of serine proteases and homologs in the diamondback moth, Plutella xylostella (L.)
Source: BMC Genomics. 2015 Dec 10;16:1054. doi: 10.1186/s12864-015-2243-4 (PMC4676143; doi:10.1186/s12864-015-2243-4)
Supplement: Additional file 6: Figure S4. — Alignment of 14 P. xylostella clip domain sequences along with the clip domains from BmSPH78, BmSPH125, MsHP6, MsHP8, MsPAP1 and MsPAP3 by Clustal X2. (PxCLIP11 has two clip domains represented by PxCLIP11a and PxCLIP11b). (DOC 352 kb) [file 12864_2015_2243_MOESM6_ESM.doc]

**
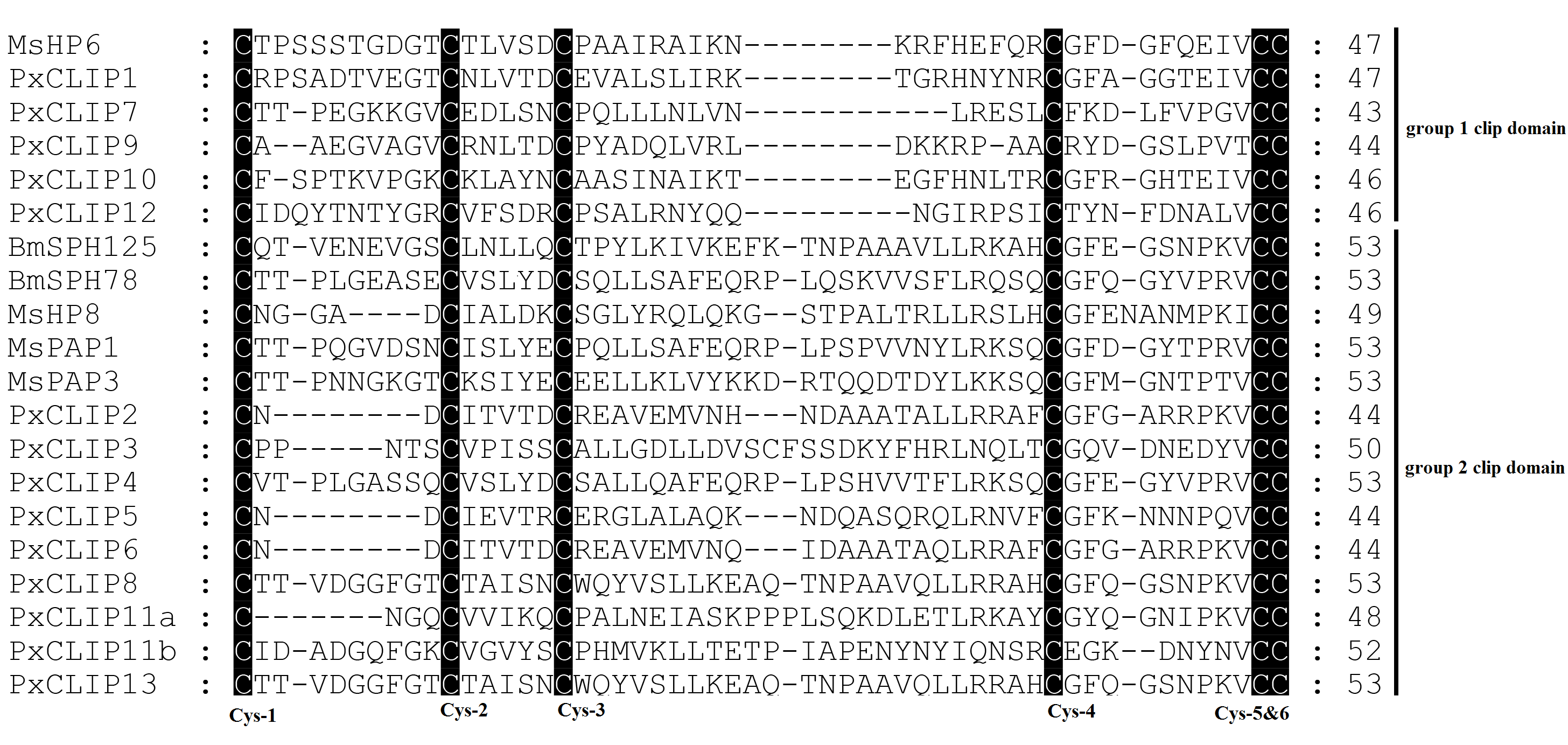
**

**Additional file 6: Figure S4.** Alignment of 14 *P. xylostella* clip domain sequences along with the clip domains from BmSPH78, BmSPH125, MsHP6, MsHP8, MsPAP1 and MsPAP3 by Clustal X2. (PxCLIP11 has two clip domains represented by PxCLIP11a and PxCLIP11b). Six conserved Cys residues are marked with black. The number represents the number of residues of a clip domain. Clip domains are usually divided into two groups depending on the number of residues between Cys-3 and Cys-4, with group 1 clip domain having 8-17 residues and group 2 having 22-26 residues [41].
