# Supplementary material for: Genome-wide identification and expression profiling of serine proteases and homologs in the diamondback moth, Plutella xylostella (L.)
Source: BMC Genomics. 2015 Dec 10;16:1054. doi: 10.1186/s12864-015-2243-4 (PMC4676143; doi:10.1186/s12864-015-2243-4)
Supplement: Additional file 7: Figure S5. — Multiple alignment of P. xylostella Nudel gene along with other three Lepidoptera Nudels: Papilio Xuthus Nudel, PaxNudel (XP_013165117.1), Papilio polytes Nudel, PpNudel (XP_013138700.1) and Amyelois transitella Nudel, AtNudel (XP_013190169.1) by Clustal X2. (DOC 2247 kb) [file 12864_2015_2243_MOESM7_ESM.doc]

**
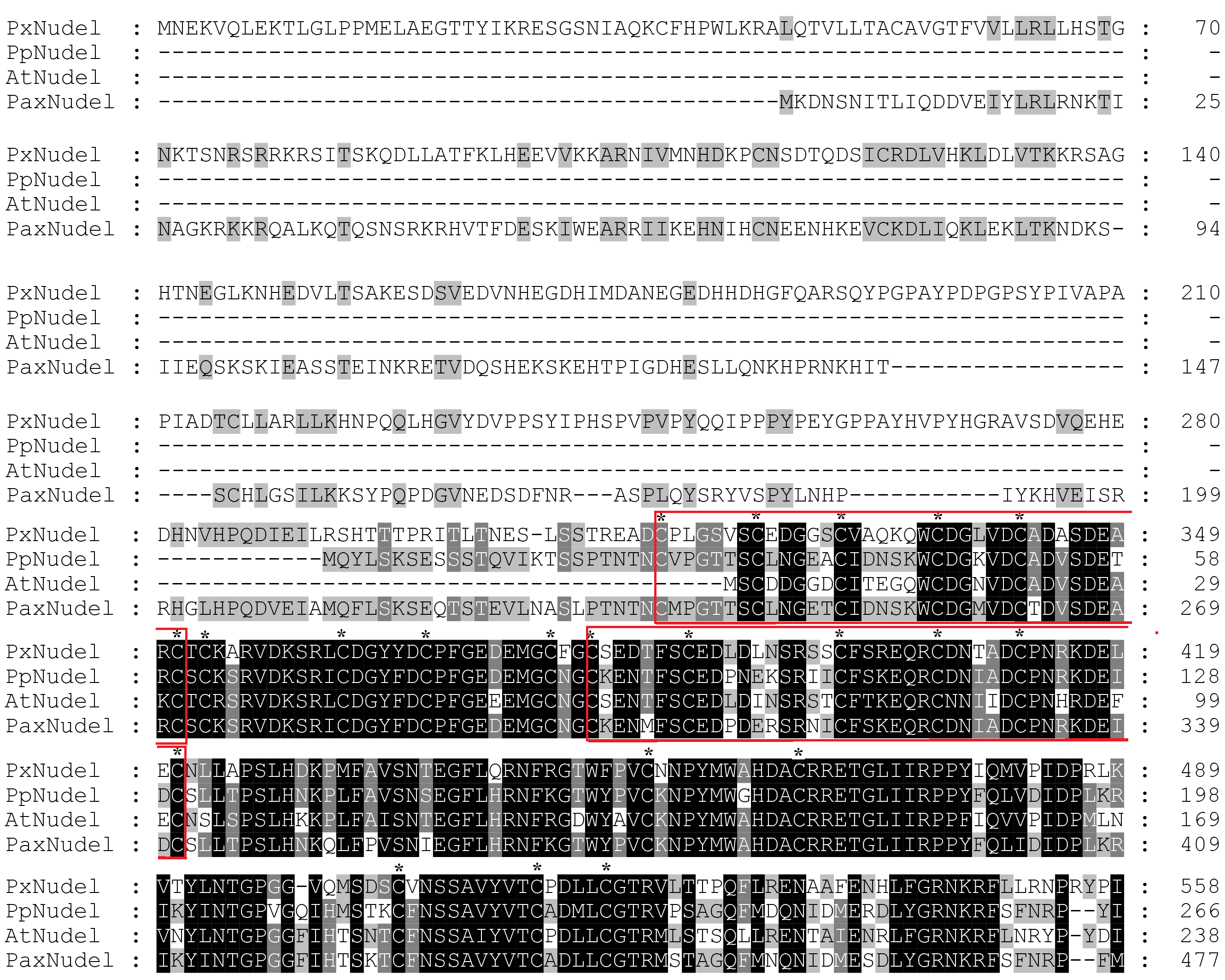
**

**
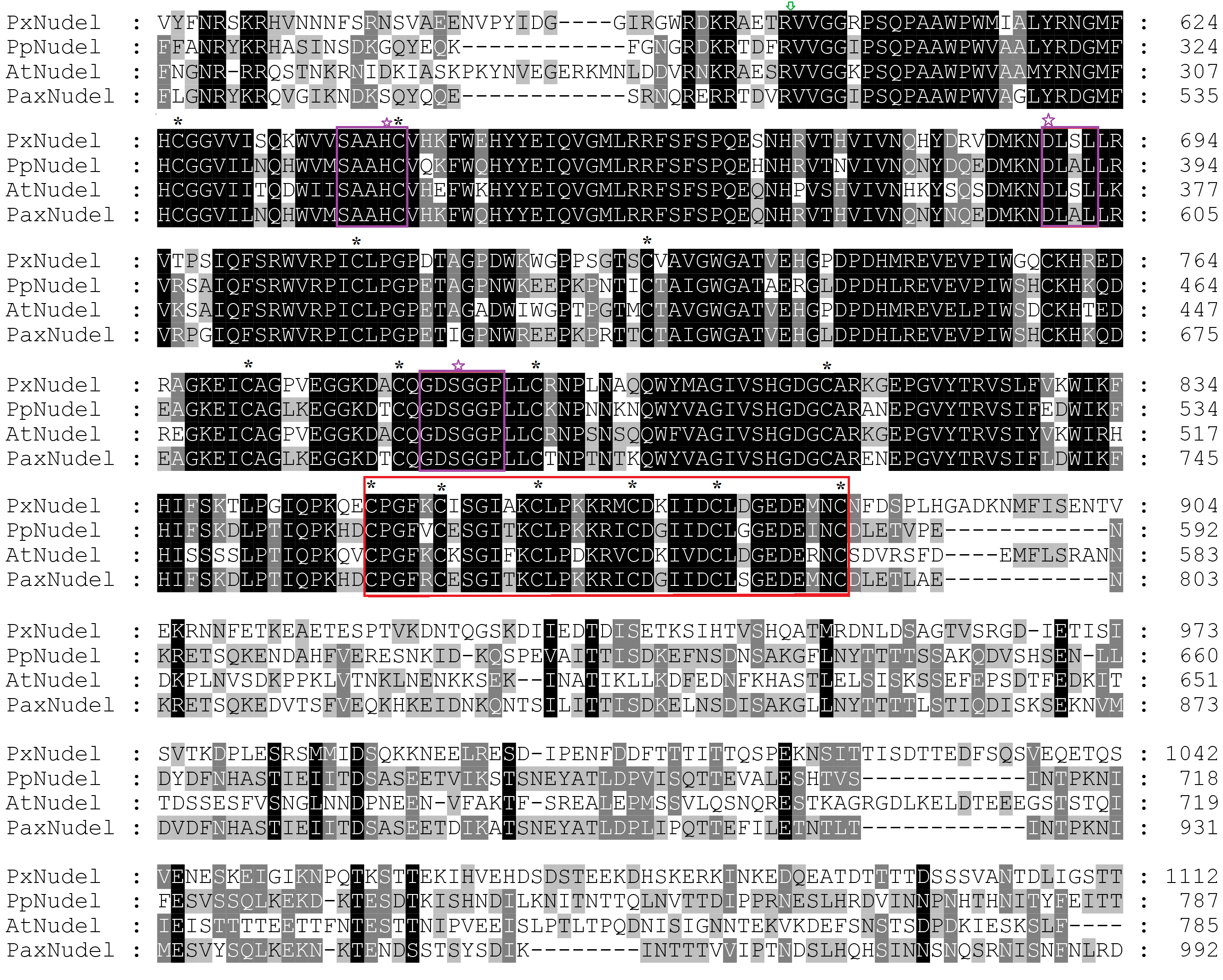
**

**
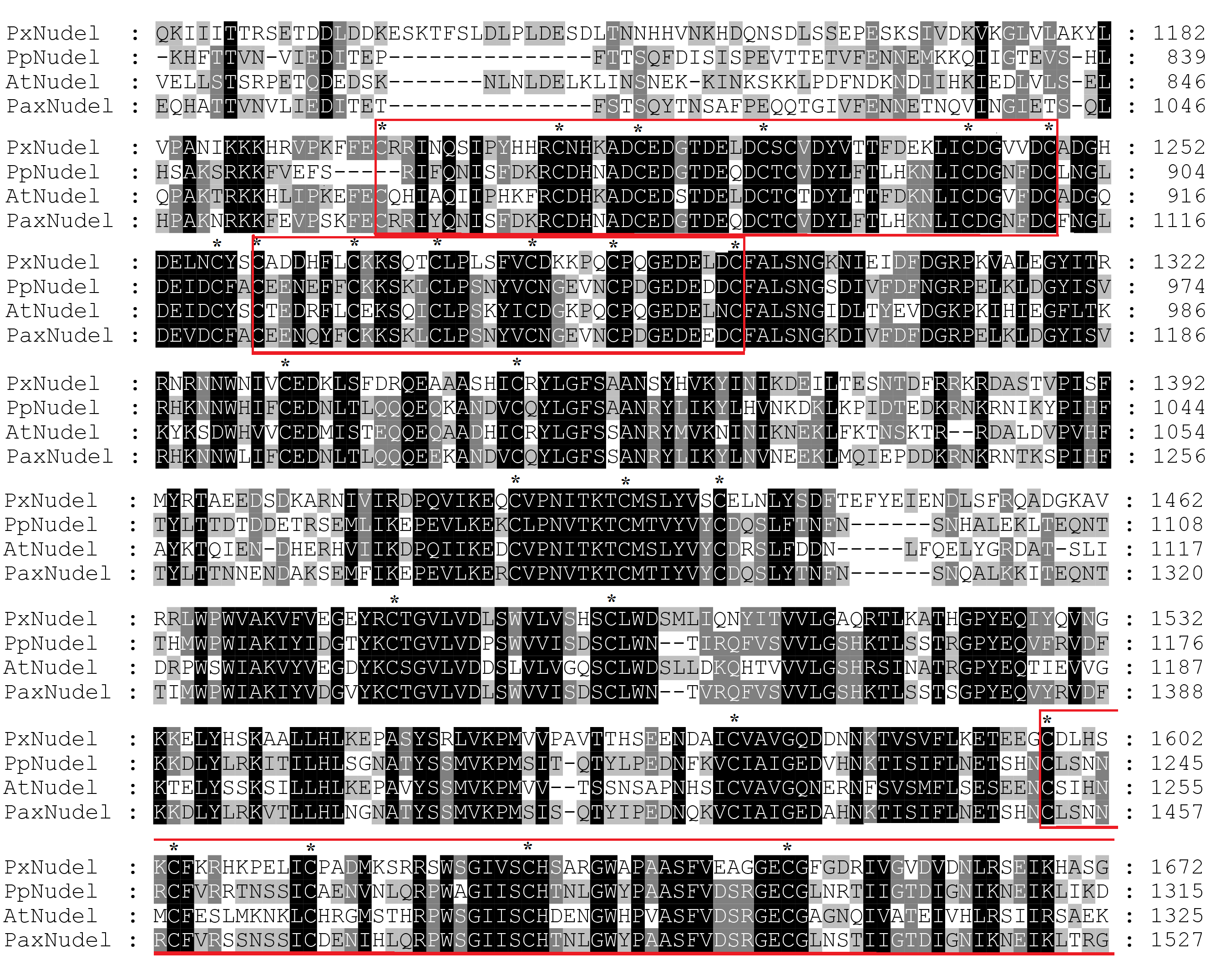
**

**
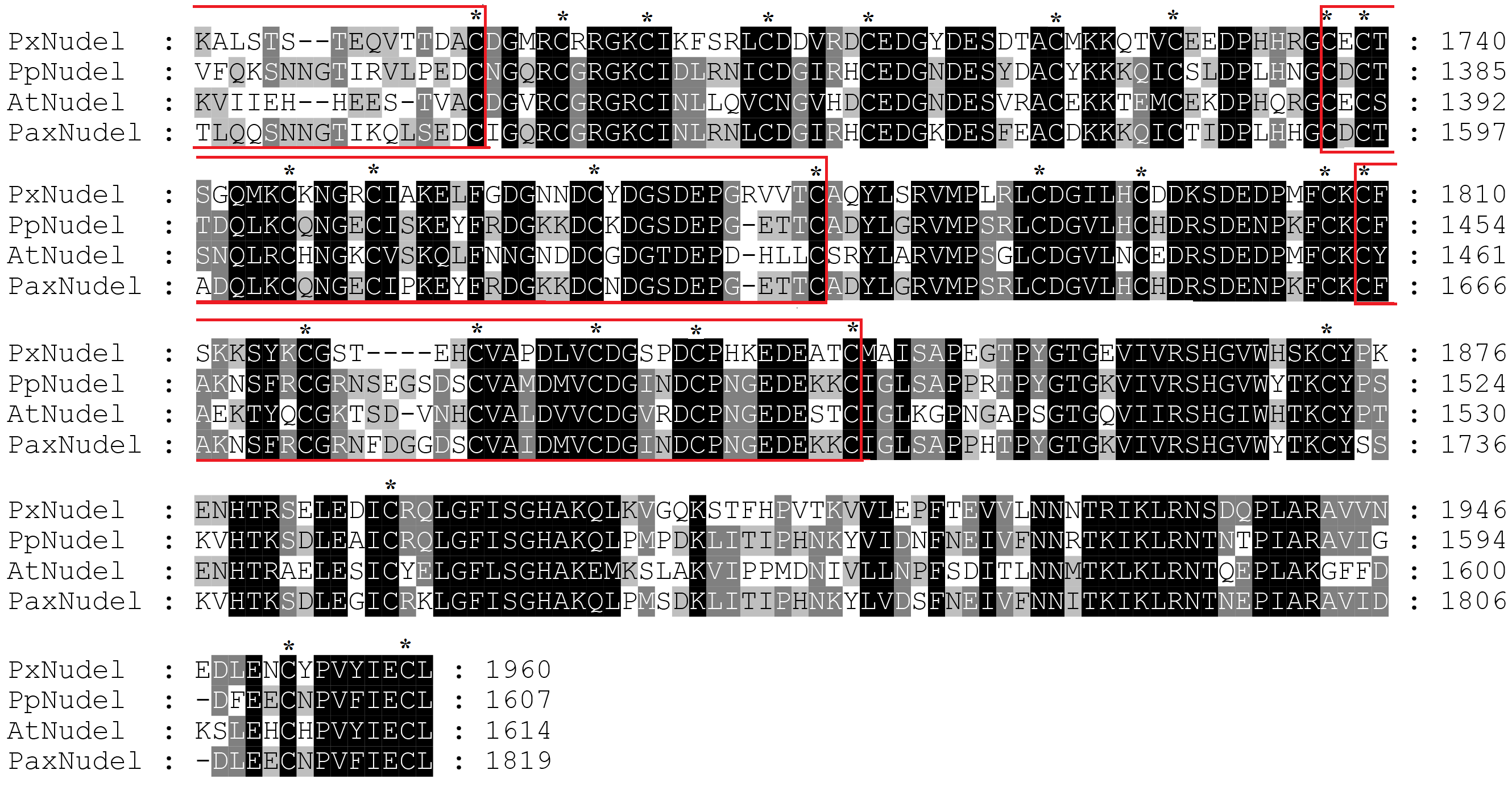
**

**Additional file 7: Figure S5.** Multiple alignment of *P*. *xylostella* Nudel gene along with other three Lepidoptera Nudels: *Papilio Xuthus* Nudel, PaxNudel (XP_013165117.1), *Papilio polytes* Nudel, PpNudel (XP_013138700.1) and *Amyelois transitella* Nudel, AtNudel (XP_013190169.1) by Clustal X2. The catalytic triads (His, Asp and Ser) are marked with purple star at the top, three conserved regions (SAAHC, DLSL, and GDSGGP) are boxed in purple, the putative autocatalytic site is marked with a green arrow at the top. Eight LDLA domains are boxed in red. Cysteine residues are marked with black stars at the top.
