# Supplementary material for: Genome-wide identification and expression profiling of serine proteases and homologs in the diamondback moth, Plutella xylostella (L.)
Source: BMC Genomics. 2015 Dec 10;16:1054. doi: 10.1186/s12864-015-2243-4 (PMC4676143; doi:10.1186/s12864-015-2243-4)
Supplement: Additional file 8: Figure S6. — Multiple alignment of P. xylostella Gd gene along with other insect species Gds, Nilaparvata lugens Gd, NlGd (AID60301.1); Apis mellifera Gd, AmGd (XP_006563318.1); Bombyx mori Gd, BmGd (XP_012548092.1); Drosophila melanogaster Gd, DmGd (ABG02140.1); Apis florea Gd, AfGd (XP_003690498.1); Nasonia vitripennis Gd, NvGd (XP_003427708.1), and Megachile rotundata Gd, MrGd (XP_012143735.1) by Clustal X2. (DOC 1166 kb) [file 12864_2015_2243_MOESM8_ESM.doc]

**
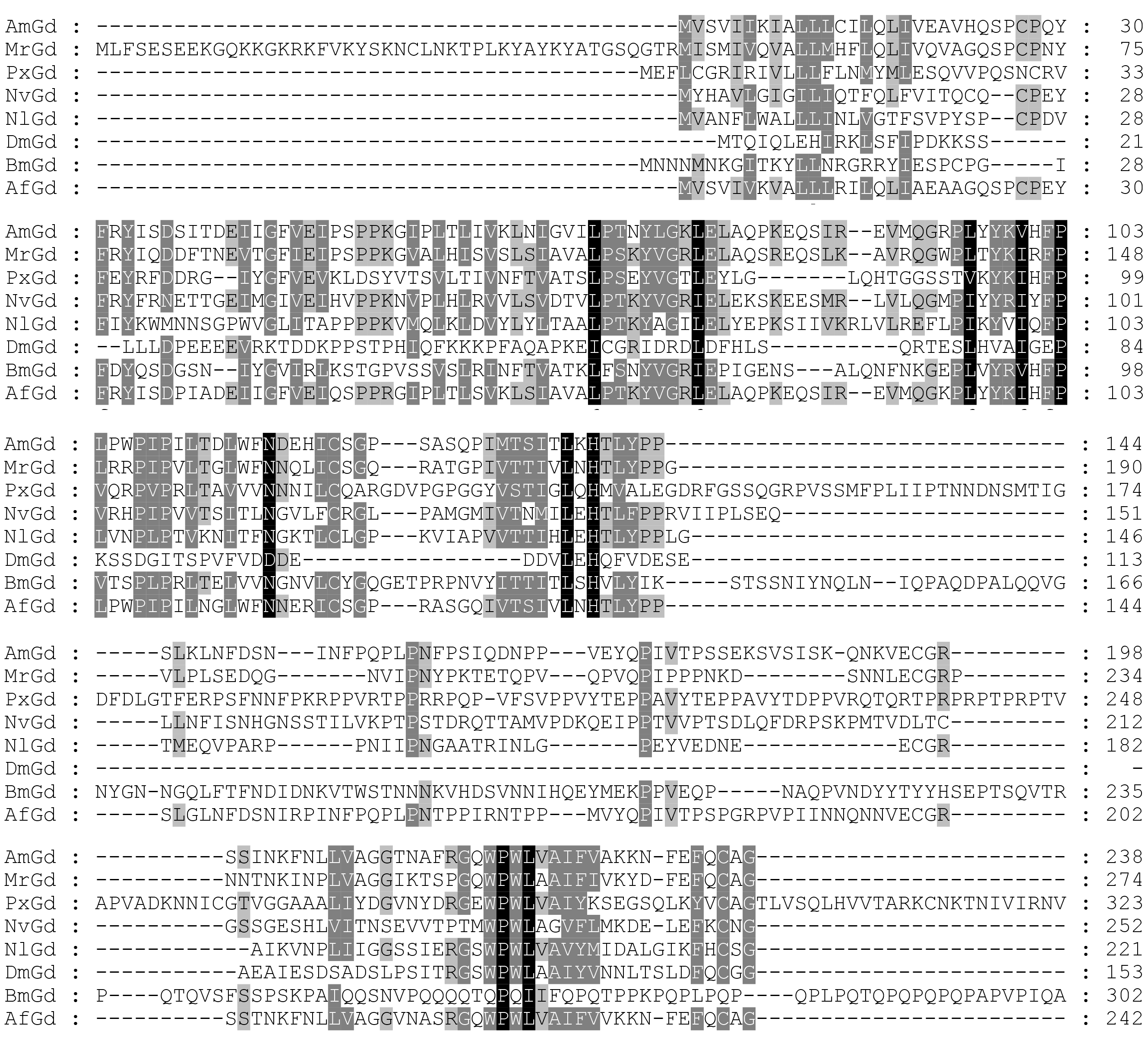
**

**
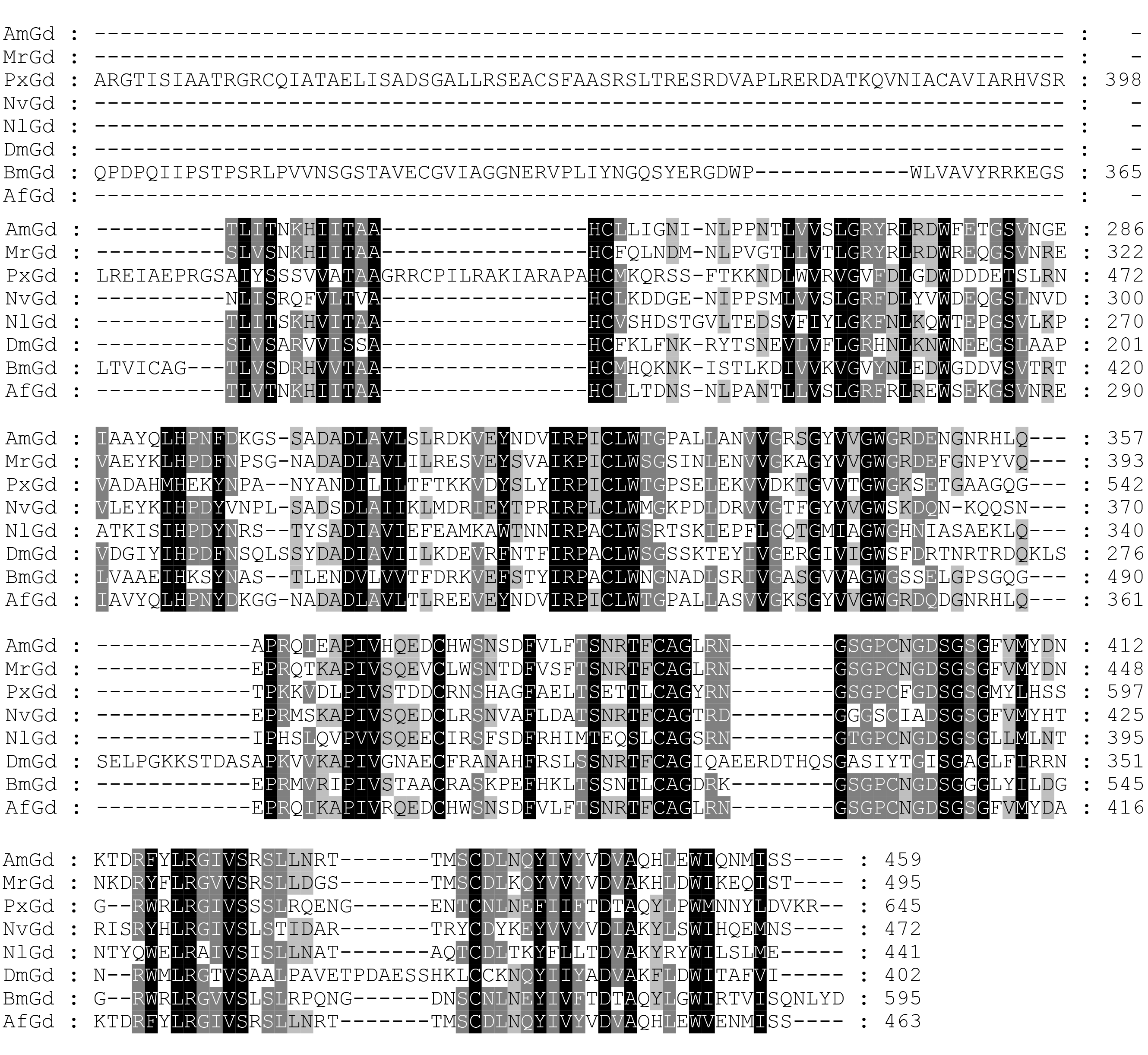
**

**Additional file 8: Figure S6.** Multiple alignment of *P*. *xylostella* Gd gene along with other insect species Gds, *Nilaparvata lugens* Gd, NlGd (AID60301.1); *Apis mellifera* Gd, AmGd (XP_006563318.1); *Bombyx mori* Gd, BmGd (XP_012548092.1); *Drosophila melanogaster* Gd, DmGd (ABG02140.1); *Apis florea* Gd, AfGd (XP_003690498.1); *Nasonia vitripennis* Gd, NvGd (XP_003427708.1), and *Megachile rotundata* Gd, MrGd (XP_012143735.1) by Clustal X2.
