# Supplementary material for: Genome-wide identification and expression profiling of serine proteases and homologs in the diamondback moth, Plutella xylostella (L.)
Source: BMC Genomics. 2015 Dec 10;16:1054. doi: 10.1186/s12864-015-2243-4 (PMC4676143; doi:10.1186/s12864-015-2243-4)
Supplement: Additional file 9: Figure S7. — Phylogenetic tree/analysis of the stubble genes in P. xylostella and other five insect species. (DOC 98 kb) [file 12864_2015_2243_MOESM9_ESM.doc]

**
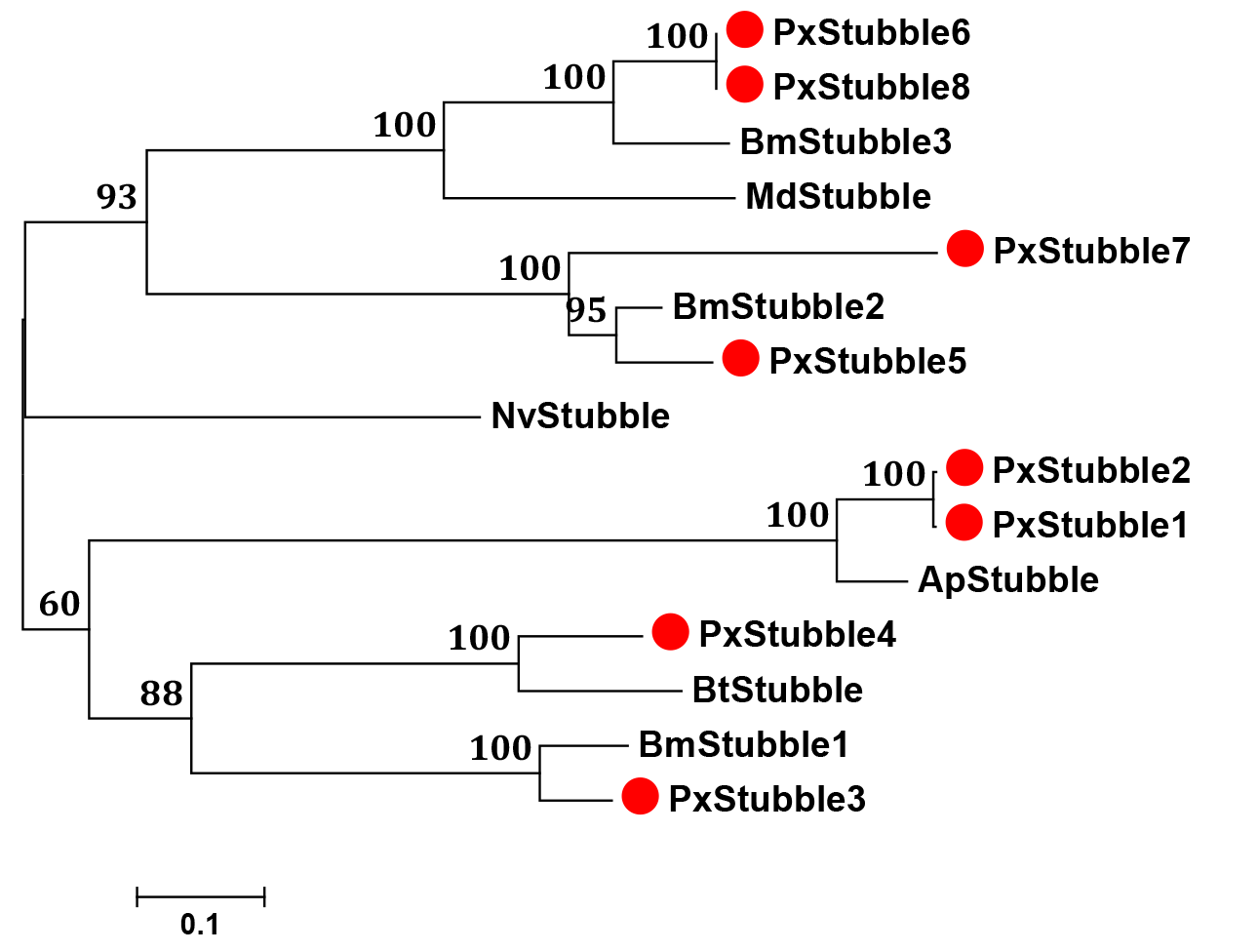
**

**Additional file 9: Figure S7.** Phylogenetic tree/analysis of the stubble genes in *P. xylostella* and other five insect species. GenBank (NCBI) accession numbers for the sequences of different species are as follows: XP_012171119 (BtStubble, *Bombus terrestris*); XP_001949227 (ApStubble, *Acyrthosiphon pisum*); XP_008214111 (NvStubble, *Nasonia vitripennis*); XP_005176542 (MdStubble, *Musca domestica*); XP_004932722 (BmStubble1, *B. mori*); XP_004933267 (BmStubble2); XP_004933266 (BmStubble3); Px004167 (PxStubble1, *Plutella xylostella*); Px004168 (PxStubble2, *Plutella xylostella*); Px005947 (PxStubble3); Px010313 (PxStubble4); Px011097 (PxStubble5); Px013864 (PxStubble6); Px013865 (PxStubble7) and Px017504 (PxStubble8).
