# Supplementary material for: Genome-wide identification and expression profiling of serine proteases and homologs in the diamondback moth, Plutella xylostella (L.)
Source: BMC Genomics. 2015 Dec 10;16:1054. doi: 10.1186/s12864-015-2243-4 (PMC4676143; doi:10.1186/s12864-015-2243-4)
Supplement: Additional file 11: Figure S8. — qPCR-based expression profiling of the SP and SPH genes across different developmental stages. (DOC 232 kb) [file 12864_2015_2243_MOESM11_ESM.doc]

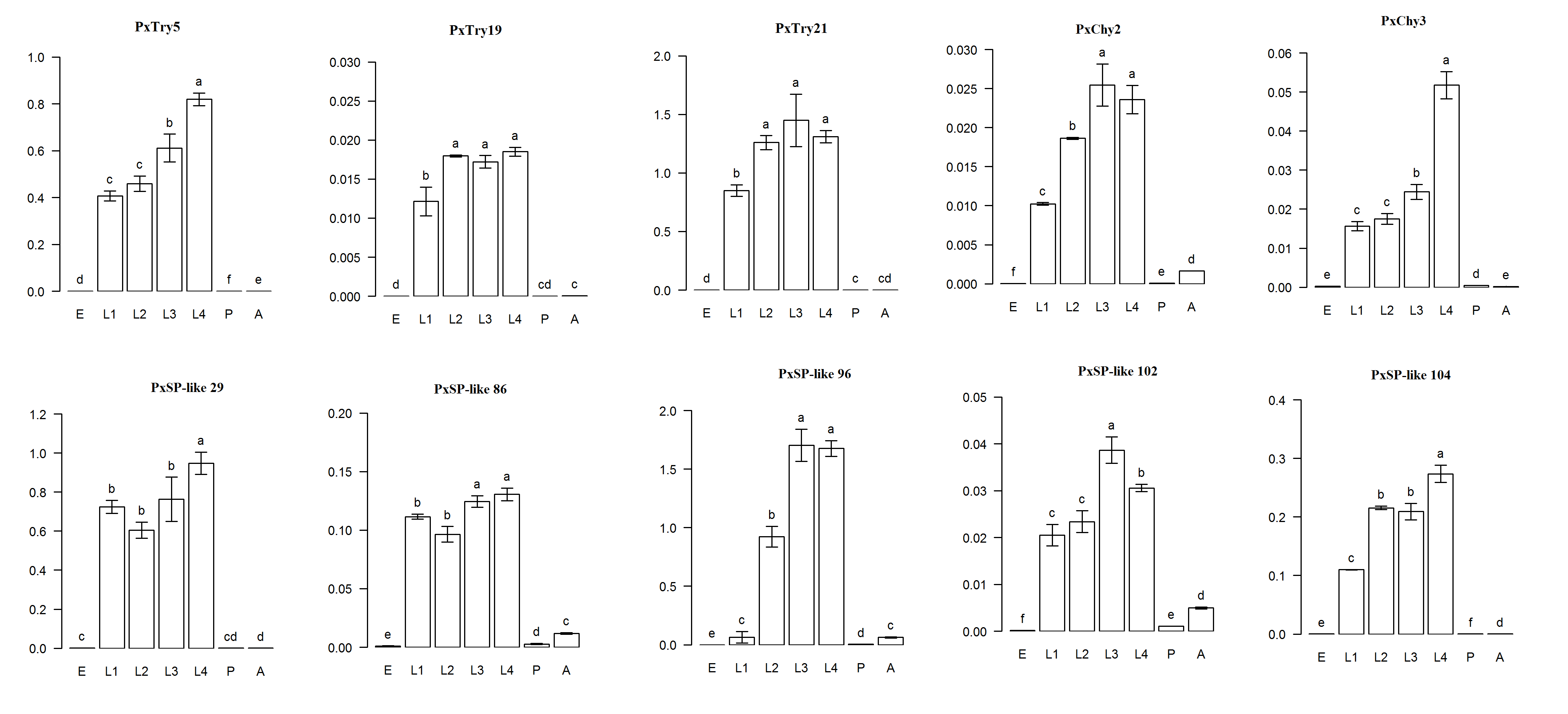


**Additional file 11: Figure S8.** qPCR-based expression profiling of the SP and SPH genes across different developmental stages. E, eggs; L1, 1^st^-instar larvae; L2, 2^nd^-instar larvae; L3, 3^rd^-instar larvae; L4, 4^th^-instar larvae; P, pupae; A, adults. X axis: samples; Y axis: relative expression value.
