# Supplementary material for: Genome-wide identification and expression profiling of serine proteases and homologs in the diamondback moth, Plutella xylostella (L.)
Source: BMC Genomics. 2015 Dec 10;16:1054. doi: 10.1186/s12864-015-2243-4 (PMC4676143; doi:10.1186/s12864-015-2243-4)
Supplement: Additional file 12: Figure S9. — Illustration for expression profiling of the P. xylostella SP and SPH genes in different tissues, showing the hierarchical clustered groups of expression pattern. (DOC 142 kb) [file 12864_2015_2243_MOESM12_ESM.doc]

**
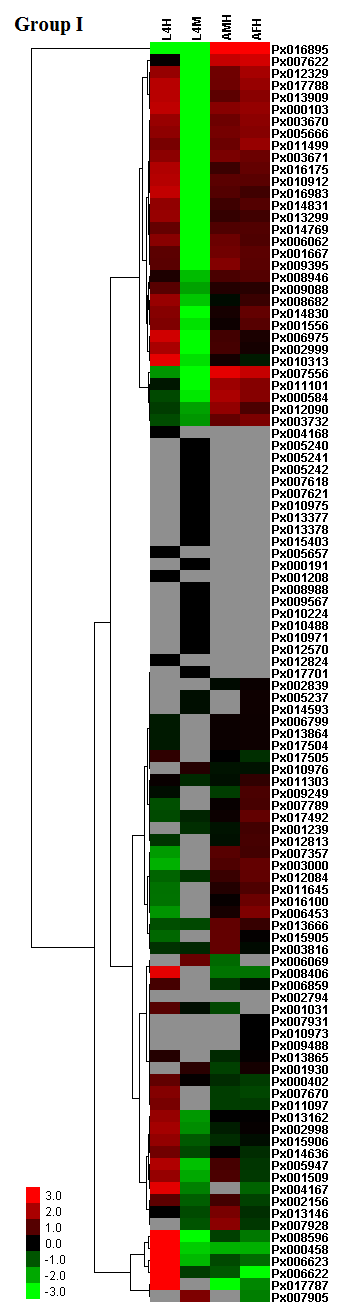

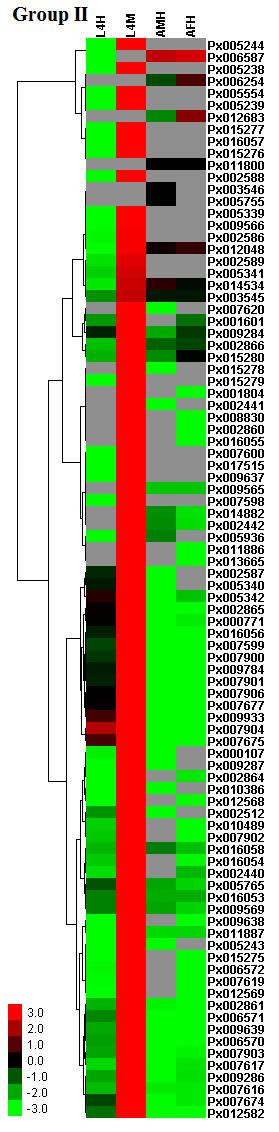
**

**Additional file 12: Figure S9.** Illustration for expression profiling of the *P. xylostella* SP and SPH genes in different tissues, showing the hierarchical clustered groups of expression pattern. The log_2_ RPKM values are presented by bar colors where the darker red represents higher expression values, the darker green represents lower expression values, and the gray represents missing values. L4M, midguts of 4^th^-instar larvae; L4H, heads of 4^th^-instar larvae; AMH, heads of adult males; FMH, heads of adult females. The RPKM values are given in Additional file 13: Table S4.
